# Evolutionary conservation and divergence of phagocytic and coagulation programs across bilaterian circulating immune cells

**DOI:** 10.1093/molbev/msag109

**Published:** 2026-04-27

**Authors:** Yanan Li, Xiang Liu, Hongxi Chen, Qi Yang, Qun Liu, Hong-Yan Wang, Shuo Li, Xianghui Zhang, Yingyi Huang, Jian-Yang Chen, Lucas B Doretto, Ivana F Rosa, Shenglei Han, Chen Li, Inge Seim, Yifang Lu, Kaiqiang Liu, Junqiang Xu, Yingying Zhang, Shijie Hao, Sha Liao, Shanshan Pan, Junjie Shi, Yadong Chen, Chenghua Li, Qian Wang, Shanshan Liu, Guangyi Fan, Changwei Shao

**Affiliations:** State Key Laboratory of Mariculture Biobreeding and Sustainable Goods, Yellow Sea Fisheries Research Institute, Chinese Academy of Fishery Sciences, Qingdao, Shandong 266071, China; Laboratory for Marine Fisheries Science and Food Production Processes, Qingdao Marine Science and Technology Center, Qingdao, Shandong 266237, China; College of Fisheries and Life Science, Shanghai Ocean University, Shanghai 201306, China; State Key Laboratory of Mariculture Biobreeding and Sustainable Goods, Yellow Sea Fisheries Research Institute, Chinese Academy of Fishery Sciences, Qingdao, Shandong 266071, China; Laboratory for Marine Fisheries Science and Food Production Processes, Qingdao Marine Science and Technology Center, Qingdao, Shandong 266237, China; Qingdao-Europe Advanced Institute for Life Sciences, BGI Research, Qingdao 266555, China; Department of Biology, University of Copenhagen, Copenhagen 2100, Denmark; BGI Research, Sanya 572025, China; Hainan Technology Innovation Center for Marine Biological Resources Utilization (Preparatory Period), BGI Research, Sanya 572025, China; State Key Laboratory of Mariculture Biobreeding and Sustainable Goods, Yellow Sea Fisheries Research Institute, Chinese Academy of Fishery Sciences, Qingdao, Shandong 266071, China; Laboratory for Marine Fisheries Science and Food Production Processes, Qingdao Marine Science and Technology Center, Qingdao, Shandong 266237, China; Department of Biology, University of Copenhagen, Copenhagen 2100, Denmark; BGI Research, Qingdao 266555, China; Qingdao Key Laboratory of Marine Genomics, BGI Research, Qingdao 266555, China; State Key Laboratory of Mariculture Biobreeding and Sustainable Goods, Yellow Sea Fisheries Research Institute, Chinese Academy of Fishery Sciences, Qingdao, Shandong 266071, China; Laboratory for Marine Fisheries Science and Food Production Processes, Qingdao Marine Science and Technology Center, Qingdao, Shandong 266237, China; State Key Laboratory of Mariculture Biobreeding and Sustainable Goods, Yellow Sea Fisheries Research Institute, Chinese Academy of Fishery Sciences, Qingdao, Shandong 266071, China; Laboratory for Marine Fisheries Science and Food Production Processes, Qingdao Marine Science and Technology Center, Qingdao, Shandong 266237, China; State Key Laboratory of Mariculture Biobreeding and Sustainable Goods, Yellow Sea Fisheries Research Institute, Chinese Academy of Fishery Sciences, Qingdao, Shandong 266071, China; Laboratory for Marine Fisheries Science and Food Production Processes, Qingdao Marine Science and Technology Center, Qingdao, Shandong 266237, China; State Key Laboratory of Mariculture Biobreeding and Sustainable Goods, Yellow Sea Fisheries Research Institute, Chinese Academy of Fishery Sciences, Qingdao, Shandong 266071, China; Laboratory for Marine Fisheries Science and Food Production Processes, Qingdao Marine Science and Technology Center, Qingdao, Shandong 266237, China; BGI Research, Qingdao 266555, China; Qingdao Key Laboratory of Marine Genomics, BGI Research, Qingdao 266555, China; State Key Laboratory of Mariculture Biobreeding and Sustainable Goods, Yellow Sea Fisheries Research Institute, Chinese Academy of Fishery Sciences, Qingdao, Shandong 266071, China; Laboratory for Marine Fisheries Science and Food Production Processes, Qingdao Marine Science and Technology Center, Qingdao, Shandong 266237, China; Department of Structural and Functional Biology, Institute of Biosciences, São Paulo State University (UNESP), Botucatu 01049-010, Brazil; State Key Laboratory of Mariculture Biobreeding and Sustainable Goods, Yellow Sea Fisheries Research Institute, Chinese Academy of Fishery Sciences, Qingdao, Shandong 266071, China; Laboratory for Marine Fisheries Science and Food Production Processes, Qingdao Marine Science and Technology Center, Qingdao, Shandong 266237, China; State Key Laboratory of Mariculture Biobreeding and Sustainable Goods, Yellow Sea Fisheries Research Institute, Chinese Academy of Fishery Sciences, Qingdao, Shandong 266071, China; Laboratory for Marine Fisheries Science and Food Production Processes, Qingdao Marine Science and Technology Center, Qingdao, Shandong 266237, China; Marine Mammal and Marine Bioacoustics Laboratory, Institute of Deep-sea Science and Engineering, Chinese Academy of Sciences, Sanya 572000, China; State Key Laboratory of Mariculture Biobreeding and Sustainable Goods, Yellow Sea Fisheries Research Institute, Chinese Academy of Fishery Sciences, Qingdao, Shandong 266071, China; Laboratory for Marine Fisheries Science and Food Production Processes, Qingdao Marine Science and Technology Center, Qingdao, Shandong 266237, China; State Key Laboratory of Mariculture Biobreeding and Sustainable Goods, Yellow Sea Fisheries Research Institute, Chinese Academy of Fishery Sciences, Qingdao, Shandong 266071, China; Laboratory for Marine Fisheries Science and Food Production Processes, Qingdao Marine Science and Technology Center, Qingdao, Shandong 266237, China; BGI Research, Shenzhen 518083, China; BGI Research, Sanya 572025, China; Hainan Technology Innovation Center for Marine Biological Resources Utilization (Preparatory Period), BGI Research, Sanya 572025, China; BGI Research, Shenzhen 518083, China; BGI Research, Shenzhen 518083, China; BGI Research, Qingdao 266555, China; Qingdao Key Laboratory of Marine Genomics, BGI Research, Qingdao 266555, China; BGI Research, Qingdao 266555, China; Qingdao Key Laboratory of Marine Genomics, BGI Research, Qingdao 266555, China; BGI Research, Qingdao 266555, China; Qingdao Key Laboratory of Marine Genomics, BGI Research, Qingdao 266555, China; School of Marine Sciences, Ningbo University, Ningbo, Zhejiang 315211, China; State Key Laboratory of Mariculture Biobreeding and Sustainable Goods, Yellow Sea Fisheries Research Institute, Chinese Academy of Fishery Sciences, Qingdao, Shandong 266071, China; Laboratory for Marine Fisheries Science and Food Production Processes, Qingdao Marine Science and Technology Center, Qingdao, Shandong 266237, China; MGI Tech, Shenzhen 518083, China; BGI Research, Qingdao 266555, China; Qingdao Key Laboratory of Marine Genomics, BGI Research, Qingdao 266555, China; BGI Research, Shenzhen 518083, China; State Key Laboratory of Mariculture Biobreeding and Sustainable Goods, Yellow Sea Fisheries Research Institute, Chinese Academy of Fishery Sciences, Qingdao, Shandong 266071, China; Laboratory for Marine Fisheries Science and Food Production Processes, Qingdao Marine Science and Technology Center, Qingdao, Shandong 266237, China

**Keywords:** innate immunity, Bilateria, evolutionary tinkering, TFE/MiT transcription factor family

## Abstract

Innate immunity represents a foundational defense strategy across bilaterians, with phagocytosis and coagulation serving as its central effector mechanisms. However, it remains uncertain whether these mechanisms evolved from conservative ancestral regulatory modules or emerged through lineage-specific adaptations. This ambiguity currently impedes a deeper understanding of immune system evolution. We constructed a cross-phylum single-cell atlas of circulating immune cells from nine bilaterian species. Our comparative analysis revealed a pattern of evolutionary tinkering, wherein core functional modules followed largely independent evolutionary trajectories. The specific phagocytic-like cells (PLCs) identified in invertebrates share a core gene regulatory network orchestrated by the MiT/TFE transcription factor family with vertebrate myeloid cells, indicating deep homology. Notably, we identify and characterize coagulation effector cells in invertebrates for the first time. These clot-associated hemocytes demonstrate transcriptome-level convergence in the absence of a conserved regulatory framework, achieving similar functional states through lineage-specific genetic pathways. Our findings highlight the distinct evolutionary trajectories of innate immune cells, distinguishing the ancient, hardwired regulatory program of phagocytosis from the convergent, adaptive nature of coagulation. This study offers a unified single-cell perspective on the assembly and diversification of the bilaterian immune system.

## Introduction

As a universal and ancient feature of metazoans, the innate immune system exhibits extensive diversity in combating pathogens (Hartenstein 2006). Circulating cells serve as primary effectors of this system across animal lineages (Buchmann 2014). A pivotal shift in understanding the evolution of these cells came with the discovery of hematopoietic stem cell niches in invertebrate skeletons, revealing a likely deep homology between the circulatory immune cells of the major invertebrate lineages within Bilateria and their vertebrate counterparts (Lian et al. 2025 ). Reflecting this shared evolutionary origin, these cell types perform essential and conserved immune functions. They are capable of recognizing nonself entities and executing innate immunity through phagocytosis of pathogens and the initiation of coagulation cascades, processes that are vital for effective wound healing and organismal survival (Canesi et al. 2002; Jiravanichpaisal et al. 2006; Ling and Yu 2006; Liu et al. 2020). The preservation of these core mechanisms highlights a profound functional unity across the bilaterian lineage.

In vertebrates, phagocytosis is mediated by professional phagocytes of the myeloid lineage, which rely on a repertoire of pattern recognition receptors to detect foreign particles (Rabinovitch 1995; Kumagai and Akira 2010). In parallel, invertebrate bilaterians possess circulating hemocytes (HMs) that execute functionally analogous pathogen clearance (Allen and Burnett 2008; Sun et al. 2010 ; Castellanos-Martínez et al. 2014; Cima et al. 2016 ; Jiang et al. 2016 ; Li et al. 2018). Comparative transcriptomic analyses further suggest that specialized blood cell types are involved in the co-option of ancient transcriptional networks that originally governed phagocytosis in unicellular ancestors (Nagahata et al. 2022). However, the common evolutionary origin of the phagocytic lineages of protostomes and deuterostomes remains an unresolved question. Likewise, coagulation constitutes another essential innate immune response, enabling rapid wound sealing to prevent infection (Esmon et al. 2011). While vertebrates utilize dedicated thrombocytes (platelets) for hemostasis, it remains uncertain whether invertebrate bilaterians possess analogous coagulocytes driven by conserved genetic programs (Loof et al. 2010). This parallel ambiguity across both core functions raises a pivotal dichotomy: Did these specialized cell types derive from a common ancestral bilaterian cell (deep homology), or did they arise through independent functional convergence? Resolving this dichotomy is crucial for understanding the evolutionary assembly of the immune system.

Spanning hundreds of millions of years of divergence, cellular morphology offers limited insight into cell identity, which is fundamentally driven by core transcriptional programs and their key transcription factors (TFs) (Davidson et al. 2003; Davidson and Erwin 2006 ; Fu et al. 2017 ). Single-cell transcriptomics provides a unique resolution to characterize these transcriptional architectures across vast phylogenetic distances, allowing us to trace the origins of innate immune cell diversity. While cell types with common lineage ancestry share core regulatory TFs, these factors may have functionally diverged over evolution (Arendt et al. 2016). Although the regulatory programs governing immune cell specification are well-characterized in vertebrates (Iwasaki et al. 2003; Davidson and Erwin 2006; Graf and Enver 2009; Fu et al. 2017), their evolutionary trajectories in early deuterostomes (e.g. ascidians) and protostomes (e.g. mollusks and arthropods) remain poorly understood. Thus, moving beyond morphological and marker-based classifications to compare comprehensive transcriptomic profiles and conserved regulatory modules across diverse bilaterian species is essential.

Here, we present a cross-phylum single-cell atlas of circulating immune cells across nine key species, bridging the evolutionary divide from protostomes and early deuterostomes to major vertebrate lineages. By characterizing the conserved regulatory logic governing phagocytic lineages and validating the in situ molecular and spatial profiles of clot-associated HMs across highly divergent invertebrates, we reconstruct the evolutionary trajectories of these two pivotal immune functions. Ultimately, this study establishes a comprehensive framework to decipher how principles of deep homology and lineage-specific convergence have shaped the evolutionary assembly of the bilaterian innate immune system.

## Results

### Circulating immune cell landscapes across nine species

**Figure 1:**
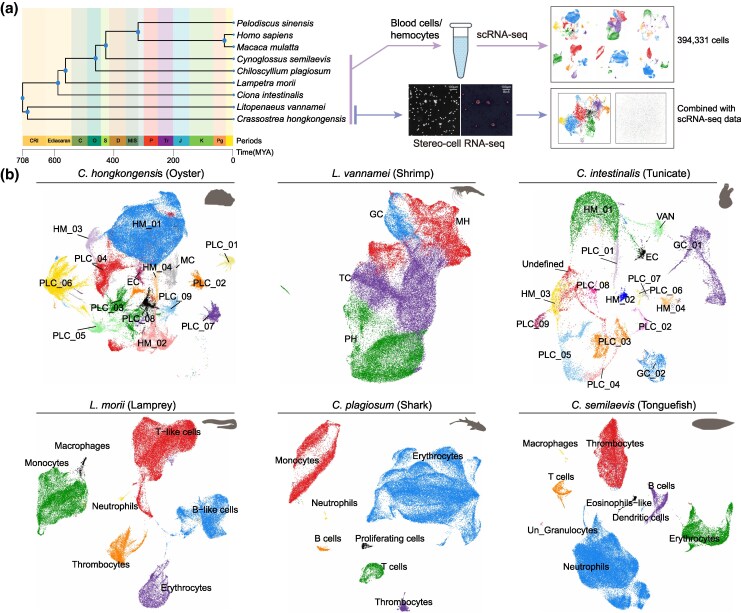
Single-cell transcriptome atlas of circulating immune cells for nine species. (a) Overall research design. Single-cell RNA sequencing was performed on hemolymph from three invertebrates (*C. hongkongensis*, *L. vannamei*, and *C. intestinalis*) and blood from three vertebrates (*L. morii*, *C. plagiosum*, and *C. semilaevis*), and integrated with publicly available data from three vertebrate species (*P. sinensis*, *M. mulatta*, and *H. sapiens*). The phylogenetic position of the species is shown. Stereo-cell RNA-seq was performed on hemolymph from *C. hongkongensis* and *L. vannamei.* The phylogenetic tree on the left was generated using the TIMETREE5 online platform (http://www.timetree.org/) (Kumar et al. 2022). (b) UMAP visualization showing cell type annotation of the six newly sequenced species: oyster, shrimp, tunicate, lamprey, shark, and tonguefish. Each cell is shown as a single dot and colored according to the assigned cluster. PLC, phagocytic-like cell; HM, non-phagocytic hemocyte; EC, epithelial cell; MC, muscle cell; VAN, vanadocyte; PH, prohemocyte; MH, monocytic hemocyte; GC, granulocyte, and TC, transitional cells.

To trace the deep evolutionary history of the circulatory immune system, we sampled nine species that span the invertebrate–vertebrate transition and occupy diverse marine, freshwater, and terrestrial habitats. The chosen species included three invertebrates [the mollusk *Crassostrea hongkongensis* (oyster), the arthropod *Litopenaeus vannamei* (shrimp), and the urochordate *Ciona intestinalis* (tunicate)] and six vertebrates [the jawless fish *Lampetra morii* (lamprey), the cartilaginous fish *Chiloscyllium plagiosum* (shark), the bony fish *Cynoglossus semilaevis* (tonguefish), the reptile *Pelodiscus sinensis* (turtle), and the mammals *Macaca mulatta* (monkey) and *Homo sapiens* (human)]. Peripheral blood single-cell data for turtles, monkeys, and humans were obtained from public repositories (see Materials and Methods), whereas fresh hemolymph or peripheral blood from the remaining species was collected to generate scRNA-seq libraries. Additionally, we applied a recently developed chip-based single-cell transcriptomic technology, Stereo-cell, to comprehensively profile the hemolymph of oysters and shrimp (Liao et al. 2025) (Fig. 1a).

Following stringent quality control and batch effect mitigation, over 440,000 high-quality single-cell transcriptomes were retained across the nine species, of which more than 390,000 were derived from six species newly generated in this study (summarized in Table S1). Unsupervised clustering and marker gene prediction revealed extensive cellular heterogeneity across all sampled species (Tables S2 to S7). Clusters were distributed evenly across all the libraries without discernible batch specificity (Figure S1a to h). Owing to the limited availability of lineage-specific molecular markers for invertebrate HMs, we annotated these cell populations based on the specific expression of known immune-related genes (Tables S8 to S10). In the oyster and tunicate, HM populations were highly heterogeneous and broadly categorized into distinct phagocytic-like cells (PLCs; defined by high expression of phagocytosis-related gene signatures), lineage-specific non-phagocytic HMs, and other specialized cells (e.g. vanadocytes and granulocytes in the tunicate) (Fig. 1b; Figure S2). The shrimp presented fewer cell types, primarily consisting of prohemocytes (PHs), monocytic hemocytes (MHs), granulocytes (GCs), and transitional cells (TCs) (Fig. 1b).

To define the PLCs in oysters and tunicates, we utilized a signature of phagocytosis-related genes, including mannose receptors, integrins, C-type lectins, scavenger receptors, and components of the phagosome-formation machinery (e.g. *RAB7, RAB20,* and *CTSL*) (Figure S3; Tables S8 and S9). Consistently, all clusters designated as PLCs exhibited significantly elevated phagocytic scores, confirming their functional identity (Figure S4a and b; Table S11). Oysters and tunicates each harbored distinct non-PLC HM clusters. These subsets were broadly characterized by the expression of lineage-specific antimicrobial effectors, homeostatic regulators, and classical HM markers (detailed in Tables S8 and S9). Furthermore, Gene Ontology (GO) enrichment analysis highlighted the functional diversity of the HM subsets, indicating their potential involvement in encapsulation, humoral secretion, and complement responses (Figures S5 and S6). For the shrimp, HMs were directly annotated using established lineage markers (Yang et al. 2022) (Figure S3; Table S10).

Among the six vertebrates, the cellular composition consisted primarily of lymphoid cells (T cells/T-like cells, B cells/B-like cells, plasma cells, and NK cells), myeloid cells (monocytes/macrophages, neutrophils, eosinophil-like cells, and dendritic cells), as well as erythrocytes and thrombocytes/platelets (Fig. 1b; Figure S7a) (Huang et al. 2024; Wang et al. 2024). The markers used for their identification are shown in Figures S3 and S7b.

### Cross-species transcriptomic mapping uncovers the deep conservation of myeloid-like innate immunity

**Figure 2:**
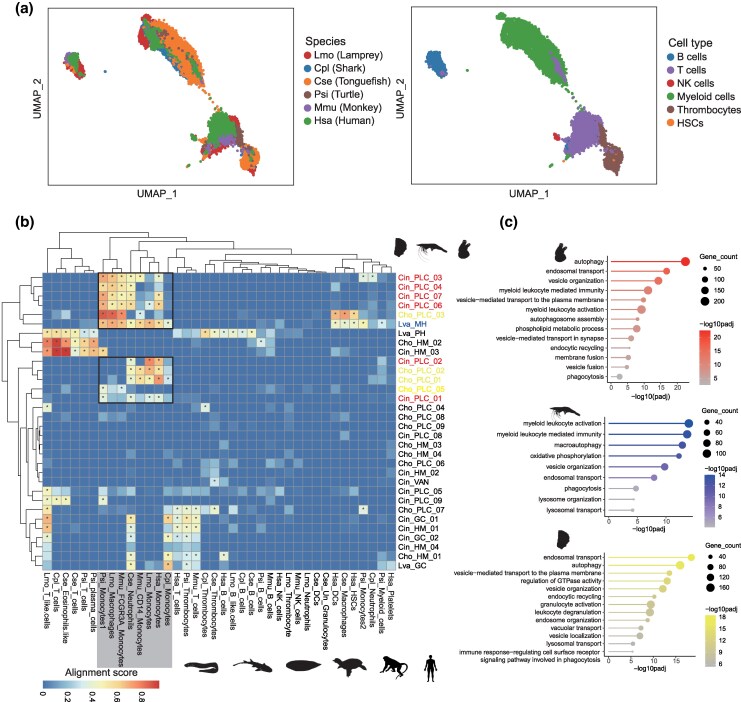
Cross species analysis of circulating immune cells in nine species. (a) UMAP projection of six vertebrate species integrated using SAMap analysis. (Left) Points are colored by species. (Right) Points are colored by cell types. (b) Heatmap of cross-species cellular similarity analyzed by SAMap, comparing hemocytes from oyster, shrimp, and tunicate with six vertebrate immune cell types. Alignment scores >0.3 are marked with asterisks (*). Black-bordered modules indicate conserved phagocytic populations. Myeloid-like phagocytes are color-coded by species: red (tunicate), blue (shrimp), and yellow (oyster). (c) GO enrichment analysis of shared homologous genes between phagocyte-like cell clusters in tunicate, shrimp, oyster and vertebrate myeloid cells.

To trace the evolutionary conservation of circulating immune cells, we systematically compared their transcriptomic profiles across the bilaterian phylogeny. Since immune cells in vertebrates share common lymphoid and myeloid origins (Boehm 2012), we first integrated the immune cell datasets from the six vertebrate species via the SAMap algorithm to establish a robust reference framework (Fig. 2a). As expected, major vertebrate lineages (including T cells, B cells, thrombocytes, and myeloid cells) exhibited highly conserved transcriptomic signatures across species. For each species, we identified differentially expressed genes (DEGs) across all immune cell types (Figure S8a). To elucidate the genetic basis of this conservation, we inferred multi-species orthologous relationships using OrthoFinder (Emms and Kelly 2019), identifying 7,138 putative orthogroups (OGs) shared among the six vertebrates (Table S12). Of these, 634 OGs contained DEGs conserved across at least five species (Figure S8b). Functional enrichment analysis showed that these genes were primarily associated with immune cell activation, differentiation, and essential innate immune processes, such as phagocytosis and coagulation, underscoring their functional conservation across different lineages of immune cells in vertebrates (Figure S8c).

To determine whether these immune programs are preserved beyond vertebrates, we compared the transcriptomic profiles of invertebrate HMs (from oysters, shrimp, and tunicates) with those of established vertebrate immune lineages. Our results revealed that specific PLC subpopulations in tunicates and oysters, alongside shrimp MHs, showed significant transcriptomic homology to vertebrate myeloid cells, specifically aligning with the monocyte/macrophage and neutrophil lineages (Fig. 2b). The orthologous genes active in these cells are fundamentally linked to ancient immune processes, including endosomal transport, vesicle organization, and phagocytosis (Fig. 2c). In contrast, certain invertebrate populations (e.g. shrimp PHs, oyster HM_02, and tunicate HM_03) also exhibited transcriptomic similarities to vertebrate lymphoid cells (Fig. 2b). However, since invertebrates lack canonical adaptive immunity (Rathinam et al. 2024), further examination of the shared orthologs revealed that this similarity is primarily driven by the conservation of basic cellular machineries (e.g. ribosome biogenesis and RNA metabolism), rather than functional immunological homology (Figure S9). Together, these comparative analyses support the deep homology of the myeloid-like innate immune program. Specifically, the ancient vesicular transport system (encompassing autophagy and endosome–lysosome pathways) and myeloid leukocyte-mediated pathogen clearance mechanisms (e.g. phagocytosis) clearly constitute the ancestral pillars of bilaterian immune defense.

### A conserved MiT/TFE-driven transcriptional program underlies phagocyte evolution across bilaterians

**Figure 3:**
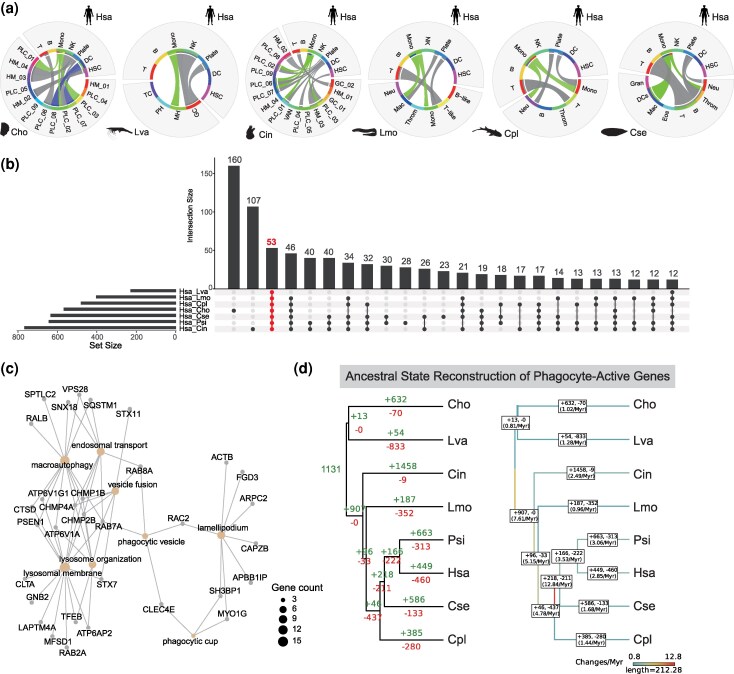
Transcriptomic homology and evolutionary dynamics of phagocytes. (a) Circos plot illustrating cross-species transcriptomic similarities between cell types of the evaluated species and human immune lineages. Arcs connecting pairs of cell types represent mappings with Kullback-Leibler Divergence (KLD)-derived similarity scores exceeding 88%, revealing a conserved transcriptomic framework across the sampled taxa. (Abbreviations: T, T cells; B, B cells; NK, NK cells; Mono, monocytes; DC, dendritic cells; Eos: eosinophils; Gran, granulocytes; HSC, hematopoietic stem cells; Mac, macrophages; Neu, neutrophils; Plate, platelets; Throm, thrombocytes). (b) UpSet plot showing the overlap of conserved orthogroups (OGs) among phagocytes from humans and the seven other analyzed species. Bar heights represent the intersection size (number of shared genes) for each species combination. (c) Functional enrichment analysis of the 53 universally conserved OGs shared across all eight species identified in (b). Orange nodes represent significantly enriched terms (*P* < 0.05), while gray nodes denote individual genes associated with these functions. These enriched modules likely represent the core biological processes underpinning conserved phagocytic functions. (d) Maximum parsimony reconstruction detailing the evolution of binary activation states for phagocyte-active OGs. Left: Green and red branches denote the evolutionary gain and loss of these OGs, respectively. Right: A heatmap-style phylogram with branches color-coded according to normalized rates of state change; edge labels specify the exact rate values. Change rates are defined as the raw number of transitions per branch normalized by the evolutionary time span of that branch (in millions of years, Myr).

Phagocytes represent an ancient immune lineage whose functional essence regarding pathogen internalization and degradation is preserved across diverse taxa. To enable systematic cross-species comparisons of gene expression profiles, we converted individual expression matrices into an “ortho-gene” matrix based on 4,455 conserved OGs (Figure S10a; Table S13; see Methods for details). We subsequently employed the Kullback-Leibler divergence (KLD) algorithm to quantify transcriptional similarity between each species and primate (human and monkey) circulating immune cells. Notably, human myeloid cell populations presented greater transcriptional similarity to phagocytic populations in other species (including PLCs in oysters and tunicates, MHs in shrimp, and myeloid cells in other animals) than non-phagocytic cell types did (Fig. 3a; Figure S10b). This conserved pattern was further validated using monkey data (Figure S10c), reinforcing the existence of shared transcriptional signatures across diverse species.

Based on the shared transcriptional landscape, we identified a core set of 53 conserved OGs consistently enriched in phagocytic populations across all species (Fig. 3b; Table S14). Functional enrichment analysis revealed that these OGs constitute the essential phagocytic machinery, including the lysosomal system (e.g. *RAB2A*, *LAPTM4A*, *TFEB*, and *GNB2*), phagocytic vesicles (e.g. *RAB8A*, *RAB7A*, and *RAC2*), phagocytic cups (*SH3BP1*, *MYO1G*, and *CLEC4E*), and lamellipodium (e.g. *APBB1IP*, *CAPZB*, *ARPC2*, and *FGD3*), collectively forming a complete pathway from pathogen recognition to internalization (Fig. 3c). To further delineate the evolutionary foundation of these programs, we performed ancestral state reconstruction (ASR), identifying 1,131 OGs putatively active in the ancestral bilaterian phagocyte (Fig. 3d; Table S15). Remarkably, this ancestral toolkit encompassed all 53 core conserved OGs, providing a robust molecular basis for the evolutionary stasis of phagocyte identity.

Crucially, we distinguished this functional conservation from its underlying regulatory control. Within the ancestral toolkit, only three ortho-transcription factors (ortho-TFs), specifically *TFEB*, *ATF3*, and *JUNB*, emerged as core regulatory drivers maintained in phagocytes across all studied species (Figure S11). While previous studies suggested that phagocytes inherit a unicellular organism program driven by CEBPα (Nagahata et al. 2022), we found that CEBPα orthologs could only be confidently identified in jawed vertebrates. In invertebrates such as oysters and shrimp, the complex evolutionary history of the CEBP family precludes a definitive CEBPα ortholog (Figure S12). This ambiguity prompted us to further investigate the core regulatory architecture of phagocytes using gene co-expression network analysis, aiming to identify a more universally conserved regulatory module across the protostome–deuterostome divide.

**Figure 4:**
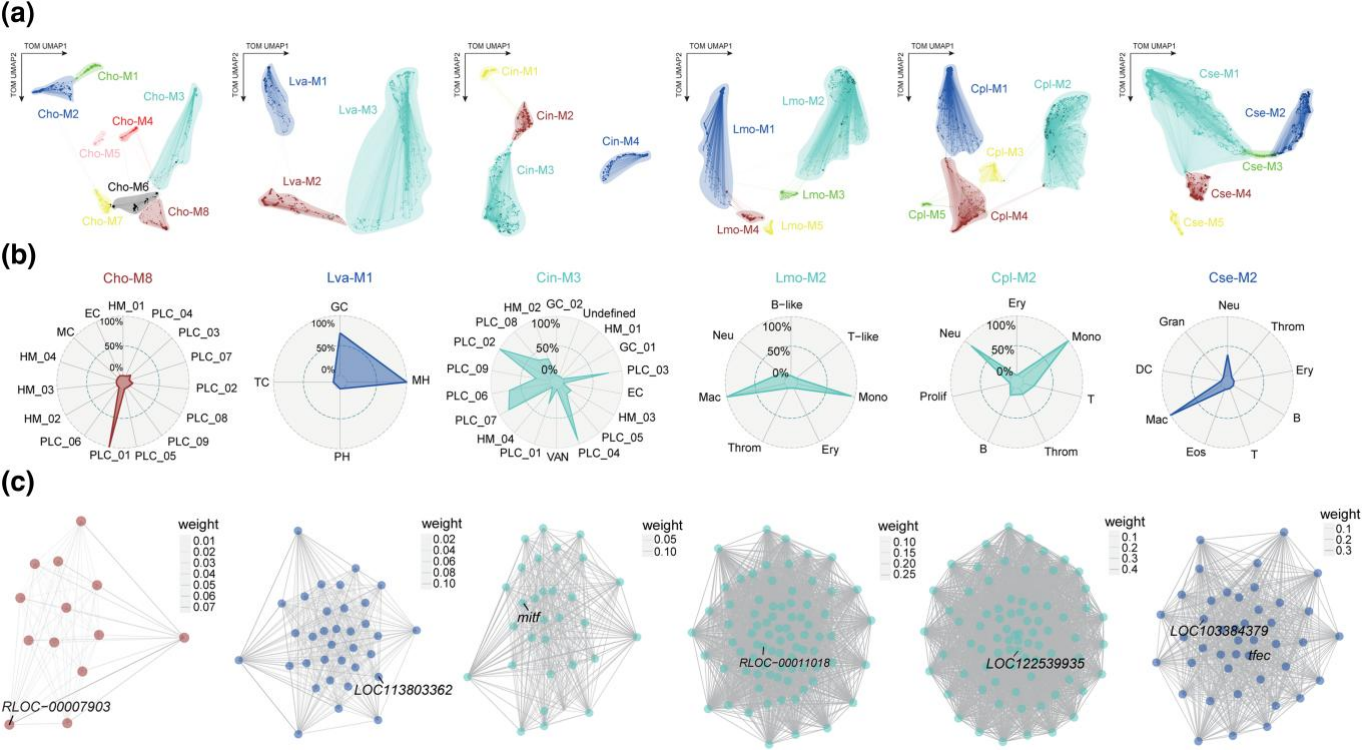
Gene co-expression networks and the conserved MiT/TFE regulatory module. (a) UMAP plot showing the gene co-expression modules identified by hdWGCNA. This analysis is based on genes from 4,455 OGs for each species. Nodes represent individual genes, and edges show significant co-expression relationships between genes and module hub genes. Node size reflects connectivity (kME values), and node color indicates the assigned module. (b) Radar plots showing the relative expression levels of selected modules across cell clusters in each species. The plotted percentage (0 to 100%) reflects the specific enrichment of these regulatory programs within phagocytic cells in each species. (c) Network plot showing the co-expression relationships involving specific members of the 53 shared OGs found in phagocytosis-specific modules across species. The MiT/TFE family transcription factors are highlighted. Each node represents a gene, and each edge shows the co-expression relationship between two genes. Line thickness reflects the strength of the relationship.

To identify a broadly conserved regulatory module across the bilaterian phylogeny, we constructed gene co-expression networks using the conserved OGs (Fig. 4a; Figure S13). This approach leverages the synchronized expression between TFs and their targets (Yu et al. 2003 ; Marco et al. 2009). Our analysis revealed a phagocyte-specific module in every studied species, consistently characterized by the presence of the MiT/TFE (*TFEB*) OG alongside specific members of the conserved 53-OG phagocytic machinery (Fig. 4b,c; Figure S13; Table S16). While vertebrates evolved 4-6 MiT/TFE paralogs through whole-genome duplications, invertebrates like oysters and shrimp maintain a single ortholog that retains a highly conserved basic helix-loop-helix-leucine zipper (bHLH-LZ) domain (Figures S14 to S16). The persistent phagocyte-specific expression and structural stasis of MiT/TFE across major evolutionary transitions suggest that phagocytes have extensively co-opted and amplified this ancient regulatory module to meet their immense demands for particle degradation and vesicular trafficking across bilaterians.

To assess the functional necessity of the MiT/TFE family in regulating the phagocytic program, we performed virtual knockout (VKO) analysis across representative taxa (Figure S17). Our results demonstrated that MiT/TFE perturbation consistently disrupted core functional modules of the phagocytic machinery. In the two invertebrates, the impact focused on fundamental trafficking mechanisms, such as vesicle organization in oysters and GTPase regulator activity in shrimp. In the tunicate, a basal chordate, the loss of MiT/TFE directly disrupted phagocytosis and autophagy. Within vertebrate branches, the MiT/TFE regulatory scope exhibited distinct functional emphases: VKO impaired myeloid homeostasis in sharks and impacted metabolic pathways in tonguefish. Notably, in amniotes (turtle and human), this regulatory scaffold was further characterized by the robust enrichment of Fc-gamma receptor signaling and lysosomal biogenesis. These findings reveal the MiT/TFE family as a conserved regulator of the phagocytic program, maintaining a core trafficking framework while recruiting lineage-specific modules to drive evolutionary diversification.

### Molecular definition of invertebrate coagulation effector cells reveals functional stasis amidst regulatory plasticity

**Figure 5:**
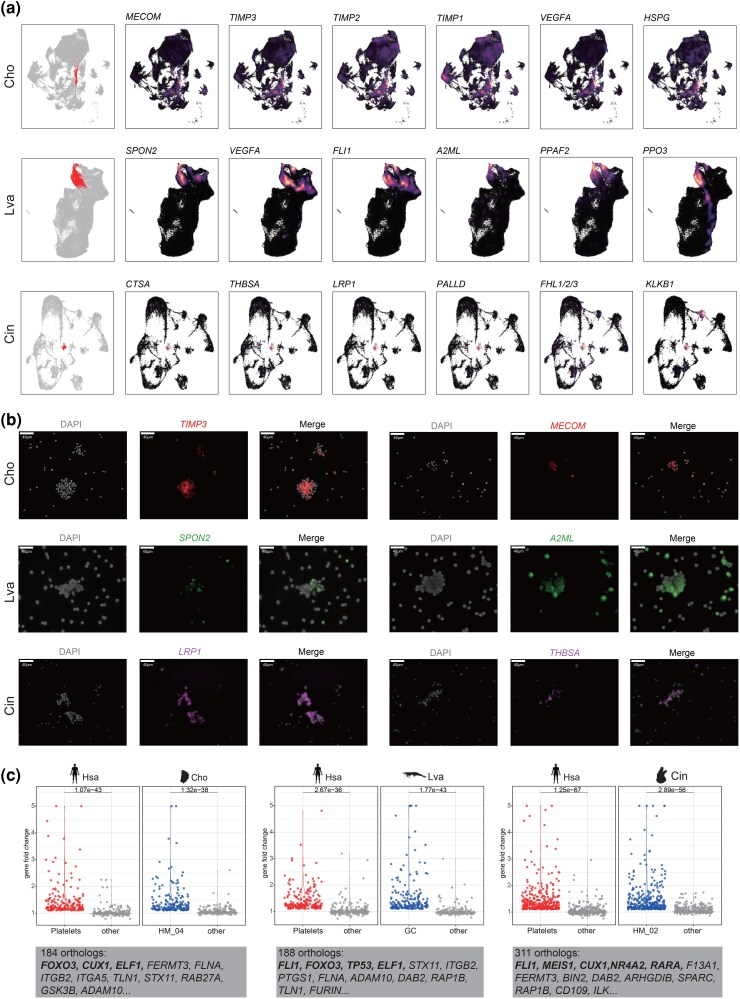
Cross-species identification and fluorescence in situ hybridization (FISH) validation of invertebrate coagulation effector cells. (a) Density plots showing expression patterns of coagulation-related genes across species. Color gradient represents expression values. (b) FISH showing the specific expression of marker genes in blood clots: *TIMP3* and *MECOM* in the oyster (red), *SPON2* and *A2ML* in the shrimp (green), and *LRP1* and *THBSA* in the tunicate (purple). Nuclei are stained with DAPI (gray). (c) Violin plots showing expression distribution of shared genes (FC > 1.1) between platelet (human) and coagulation effector cells (oyster, shrimp, and tunicate). The specific genes are shown in the gray box, and the TFs are highlighted in bold.

Coagulation, a pivotal innate immune defense mechanism in invertebrate HMs, has been observed in various invertebrates, such as oysters and shrimp (De la Ballina et al. 2022; Mengal et al. 2023). However, the molecular identity of the specific effector cells responsible for clot formation has remained elusive. We identified putative coagulocytes in oysters (HM_04), shrimp (GC), and tunicates (HM_02), characterized by the specific expression of functional effectors and regulatory homologs (Fig. 5a; Table S17). Oyster HM_04 specifically expresses TIMP family genes (*TIMP1*, *TIMP2*, and *TIMP3*), which are markedly upregulated during wound healing and inhibit MMP2 activity, suggesting their involvement in coagulation through extracellular matrix modulation (Montagnani et al. 2001 ; An et al. 2021) (Fig. 5a). These cells also highly express *MECOM*, a key TF for megakaryocyte differentiation (Shimizu et al. 2002), *VEGFA* homologs that regulate HM migration (Cho et al. 2002 ), and gene related to heparan sulfate proteoglycan (HSPG) synthesis, with HSPG experimentally verified to play a crucial role in HM aggregation (Lin et al. 2020). Shrimp GC display unique expression profiles of coagulation-related genes, including *SPON2* and *FLI1*, which are homologous to vertebrate megakaryocyte marker genes (Franzén et al. 2019 ), *A2ML*, which is involved in shrimp clot formation (Chaikeeratisak et al. 2012). Crucially, this cluster is enriched for *PPO3* and *PPAF2*, confirming their intrinsic capacity to drive biochemical coagulation and melanization through the classical proPO cascade (Theopold et al. 2004; Boonchuen et al. 2021). In tunicate HM_02, we detected multiple platelet/megakaryocyte marker homologs, including *CTSA*, *PALLD*, *FHL1/2/3*, and *LRP1* (Fig. 5a). Strikingly, these cells also express *KLKB1* (plasma kallikrein), an ancestral vertebrate-like coagulation initiator, suggesting a transitional evolutionary phenotype (Ponczek et al. 2020). Fluorescence *In Situ* Hybridization (FISH) confirmed the enrichment of *TIMP3*/*MECOM* (oyster), *SPON2*/*A2ML* (shrimp), and *LRP1*/*THBSA* (tunicate) specifically within the coagulation matrix (Fig. 5b). Notably, these three cell subpopulations showed higher transcriptional similarity compared to other HM types (Figure S18a), implying they might share similar coagulation functions. GO enrichment further supported these clusters as multifunctional hubs, showing enrichment for “platelet degranulation” and “activation” alongside broader immune responses (Figure S18b). These findings suggest an evolutionary paradigm where a single multifunctional HM lineage executes integrated hemostatic and immune functions, rather than the specialized coagulation cells found in vertebrates.

To explore the evolutionary conservation of these coagulation effector cells, we compared their transcriptional profiles with human platelets. This cross-species comparison revealed significant transcriptional similarity between the putative coagulocytes of oysters, shrimp, and tunicates with human platelets (Figure S19a), suggesting a conserved cellular identity across these distant lineages. Analysis of the orthologous gene sets co-upregulated in both invertebrate coagulation effectors and human platelets revealed a profound functional stasis; these conserved genes are predominantly involved in specialized hemostatic pathways essential for core platelet activities, including adhesion to exposed extracellular matrices (“Focal adhesion”), activation processes involving shape change and granule secretion (“signaling by Rho GTPases” and “vesicle-mediated transport”), and aggregation (“wound healing” and “hemostasis”) (Fig. 5c; Figure S19b–d).

However, a striking divergence emerged within the upstream transcriptional landscape. Despite identifying several TFs shared between individual invertebrates and humans, such as *FOXO3*, *CUX1*, and *ELF1* in oysters, no single TF was universally conserved across these three invertebrate species and humans (Fig. 5c). This lack of a universal regulator amidst high functional conservation unveils a model of regulatory plasticity. It suggests that while the functional output of coagulation cells has remained under strong purifying selection, the specific transcriptional programs defining these cells have undergone extensive independent recruitment or rewiring across different evolutionary trajectories.

### Chip-based in situ verification of coagulation effector identity in oyster and shrimp via Stereo-cell

**Figure 6:**
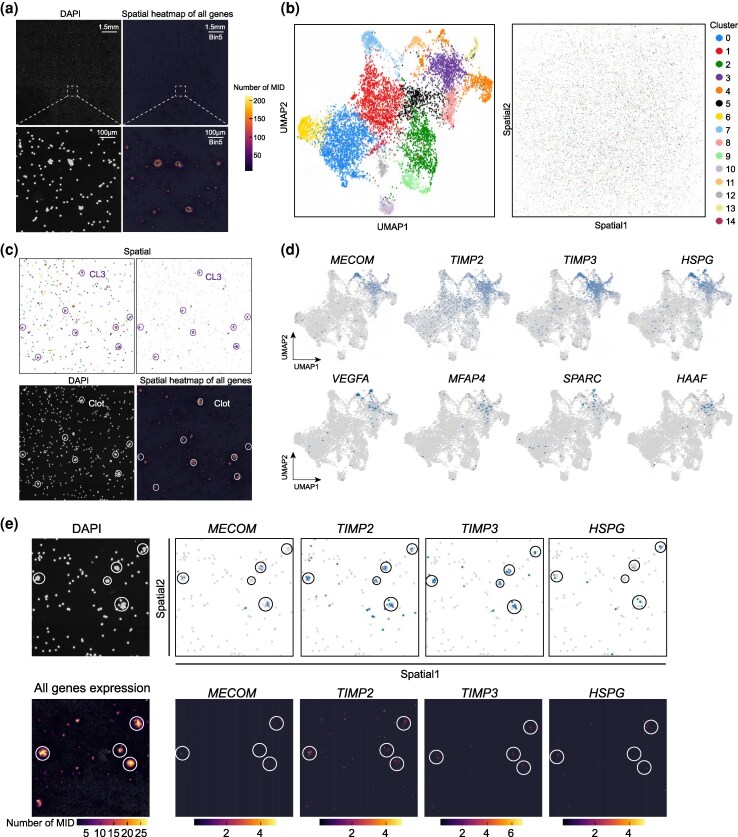
*In situ* single-cell mapping of oyster coagulation using stereo-cell technology. (a) Heatmap showing spatial gene expression at bin 5 resolution in oyster hemocytes. Nuclei were visualized using DAPI (white). Scale bar: 1.5 mm, above; 100 μm, below. (b) UMAP plot (left) and spatial visualization (right) of *C. gigas* hemocyte cell populations based on gene expression. (c) Top, spatial-resolved Cluster 3 (CL3; see B) is found adjacent to blood clots (Clot). Bottom-left, DAPI staining. Bottom-right, spatial gene expression of Cluster 3. (d) UMAP plot showing the marker genes specifically expressed in Cluster 3. (e) DAPI staining and gene heatmap display the expression information of *MECOM*, *TIMP2*, *TIMP3* and *HSPG* in Cluster 3.

To provide high-resolution evidence for the cells involved in clotting, we employed Stereo-cell, a nanoscale in situ single-cell transcriptomic technique, to analyze the hemolymph of the oyster *Crassostrea gigas* and the shrimp *L. vannamei* (Liao et al. 2025). For both species, the DNA-labeled nanoballs on the chip were binned at 5 × 5 (Fig. 6a; Figure S20a). Multiple cell clusters in the hemolymph displayed high transcriptional activity. On the chip, there were some naturally aggregated HM clusters (Fig. 6a; Figure S20a). Through unsupervised clustering of the *C. gigas* in situ dataset, we identified 15 distinct HM populations (Fig. 6b). This in situ analysis in *C. gigas* serves as a cross-species validation of the coagulation cells identity previously defined in *C. hongkongensis*, utilizing the established reference genome for precise coordinate alignment. Among these populations, Cluster 3 (CL3) was determined to be the primary component of clots, indicating a crucial role in the hemolymph clotting process (Fig. 6c). This cluster specifically expressed conserved coagulation-related genes (such as *MECOM*, *TIMP2*, *TIMP3*, and *HSPG*) that were previously identified in *C. hongkongensis* HM_04 through conventional scRNA-seq analysis (Fig. 6d). Subsequent validation using Stereo-cell confirmed the spatially restricted expression patterns of these genes within the clotting structures, underscoring their functional significance in clot formation (Fig. 6e). We also found novel features of CL3, including expression of hemagglutinin/amebocyte aggregation factor (*HAAF*) and microfibril-associated protein 4 (*MFAP4*) (Fig. 6d). Integration of the Stereo-cell and scRNA-seq datasets demonstrated strong correspondence between CL3 and HM_04 (Figure S21a). The GO terms enriched by conserved marker genes of these two cell clusters were mainly related to cell adhesion and aggregation (Figure S21b).

In shrimp, the binned data were divided into 9 different clusters, with Cluster 1 (CL1) constituting the major cellular component of clots (Figure S20b and c). This cluster exhibited expression of the same coagulation-related genes, such as *SPON2*, *VEGFA*, which was consistent with those identified in granulocytes via scRNA-seq (Figure S20d). The expression of these genes can be localized to HM clots (Figure S20e). The integration of data from the two sequencing techniques indicated that CL1 corresponded to granulocytes (Figure S20f).

To conclude, our results represent the first identification of HM subpopulations with coagulation functions in oysters and shrimp, establishing a clear cellular foundation for invertebrate coagulation and shedding new light on the evolutionary emergence of thrombocyte-like cells.

## Discussion

Our comparative single-cell atlas across major bilaterian lineages reveals that the innate immune system assembled through evolutionary tinkering (Jacob 1977), wherein core functional modules followed largely independent evolutionary trajectories. This model offers a unifying framework to interpret our principal findings: the repeated recruitment of the MiT/TFE TF network for phagocytic function across divergent lineages and the emergence of coagulation effector cells that are functionally convergent yet molecularly divergent.

The immense pressure of diverse environmental niches has sculpted the invertebrate immune system into highly plastic machinery, resulting in substantial divergence in HM lineage architecture (Raddi et al. 2020; Tattikota et al. 2020; Feng et al. 2021; Yang et al. 2022; Xin and Zhang 2023). Consequently, moving beyond traditional morphological classification to high-resolution molecular definitions is imperative for reconstructing immune cell evolution. Here, we redefine the landscape of invertebrate immunity by uncovering the level of cellular heterogeneity in oysters and tunicates that defies the traditional “limited cell type” paradigm (Sun et al. 2006; Cima et al. 2016; Rosental et al. 2018; Meng et al. 2022; Meng and Wang 2022). In our study, we uncovered multiple phagocytic cell-type clusters in oyster HMs, each exhibiting distinct transcriptional profiles and expressing a broad repertoire of phagocytic receptors. This expansion may reflect adaptations to the microbially complex intertidal zone (Saco et al. 2023), suggesting that environmental exposure to diverse pathogens could drive the functional specialization and diversification of phagocytic lineages. We speculate that the observed high phagocyte heterogeneity in oysters and tunicates likely results from a combination of intense pathogen pressure in their respective habitats and lineage-specific evolutionary trajectories favoring a diversified cellular defense strategy. In stark contrast to the extensive phagocytic diversity observed in oysters and tunicates, the shrimp HM landscape appears unexpectedly streamlined. This striking divergence raises the intriguing possibility that such cellular streamlining may represent a conserved evolutionary strategy across the broader decapod crustacean lineage (Koiwai et al. 2021; Yang et al. 2022; Li, Zhou et al. 2022b; Xin and Zhang 2023; Hou et al. 2025) rather than a species-specific anomaly.

Cross-species analysis affirms that cell types, as evolutionary units, can maintain deep homologies (Arendt et al. 2016; Wang et al. 2021; Li, Wang et al. 2022a; Mah and Dunn 2024). Phagocytosis is recognized as one of the most ancient and fundamental defense mechanisms. While previous studies have attempted to identify phagocytic cells within the circulating HMs of individual invertebrate species that share phenotypic and molecular characteristics with mammalian macrophages (Rosental et al. 2018; Tattikota et al. 2020; Yang et al. 2022), the overarching evolutionary relationship across lineages has historically remained elusive. Here, through a systematic cross-species analysis, we provide comprehensive evidence demonstrating a deep homology between invertebrate phagocyte-like cells and vertebrate myeloid phagocytes across Bilateria.

Previous studies proposed C/EBPα as an ancient phagocytic driver bridging unicellular organisms and deuterostomes (Nagahata et al. 2022). In our analyses, we traced definitive *C/EBPα* orthologs only to jawed vertebrates. Although broader C/EBP family genes exist in the examined protostomes (*C. hongkongensis* and *L. vannamei*), their profound sequence divergence, by our criteria, excluded them from the core conserved orthogroups that include vertebrate C/EBPα. To uncover the foundational regulatory architecture shared across Bilateria, our systematic network screening identified the MiT/TFE TF family as an evolutionarily conserved regulatory module in the lineages sampled. Invertebrates typically possess only a single MiT/TFE ortholog, whereas vertebrates have expanded this family via whole-genome duplication (La Spina et al. 2021), enabling functional specialization (e.g. MITF in melanocytes (Levy et al. 2006)) while preserving ancestral phagocytic functions. Gene duplication within the MiT family illustrates how such expansions facilitate functional innovation while maintaining essential host defense mechanisms. Crucially, invertebrate MiT/TFE orthologs appear to perform core phagocytic functions that are distributed across vertebrate paralogs, implying an ancient regulatory program predating vertebrate immune diversification in the bilaterian ancestor.

Phylogenetic and molecular evidence suggests that the coagulation and immune systems share a common ancestral origin, likely dating back to early bilaterian evolution (Antoniak 2018; Arneth 2019). This study resolves a long-standing paradox in hemostatic evolution: the conservation of clot formation in the absence of conserved molecular regulators. By mapping the transcriptomic signatures of these newly defined coagulation effector cells to established physiological paradigms, we reveal highly divergent, lineage-specific strategies across evolutionary distant bilaterians. Shrimp effector cells drive classical enzymatic crosslinking pathways through the specific enrichment of proPO cascade components (*PPO3* and *PPAF2*) (Theopold et al. 2004). Conversely, oyster HMs embody a matrix-driven physical plugging mechanism dominated by adhesive (*HSPG*), and extracellular matrix protective (*TIMPs*) genes (Montagnani et al. 2001; Lin et al. 2020). Furthermore, the tunicate cluster reveals a transitional proto-vertebrate state coexpressing platelet-like functional homologs and components of the intrinsic coagulation pathway (*KLKB1*) (Ponczek et al. 2020). Despite their shared capacity to form clots, they may represent a convergent “adhesive-effector” state recruited for hemostasis in different lineages. Independent evolutionary trajectories, driven by the universal imperative to rapidly seal wounds, have converged upon similar molecular functional states (Antoniak 2018; Arneth 2019). This model aligns with the known diversity of arthropod coagulation factors, which often represent novel designs rather than orthologs of vertebrate counterparts (Theopold et al. 2004). The stark contrast between conserved physiology and divergent molecular mechanisms suggests that strong selective pressures for rapid wound healing independently mold distinct genetic toolkits. We propose that this represents a form of evolutionary tinkering, where a common physiological problem is solved by co-opting different available molecular components in different lineages. In such a scenario, critical regulatory control may have shifted from transcription to other layers, such as post-translational modifications or non-coding RNAs. Ultimately, deciphering these non-orthologous paths to a common function will not only refine evolutionary theory but could also reveal new regulatory nodes with therapeutic potential for managing hemostasis.

We acknowledge that our current species sampling, while phylogenetically strategic, represents only a fraction of the vast bilaterian diversity. Furthermore, translating inferred gene regulatory networks into definitive causal mechanisms will require future functional perturbation studies in these non-model organisms. Nevertheless, the data presented here are sufficient to delineate the fundamental principles of the tinkering-based evolutionary model. Phagocytes rely on an ancient and stable regulatory core, highly conserved across bilaterians, ensuring their basic functions remain unchanged while allowing different species to develop specific receptors and adaptive mechanisms. Conversely, the core function of coagulation cells in blood clotting is achieved across different lineages through the recruitment of specific regulatory programs and molecular elements, resulting in functionally similar but molecularly distinct cell types. By distinguishing between the hardwired legacy of phagocytosis and the adaptive tinkering of coagulation, this work offers a unified perspective that fundamentally reshapes our understanding of the origins and plasticity of bilaterian immunity. Integrating these findings with emerging single-cell datasets from other key taxa (e.g. annelids, echinoderms) will be crucial for mapping the completeness of immune cell evolution and examining the universality of regulatory programs.

Collectively, our cross-phylum atlas uncovers the tinkered architecture of innate immunity, contrasting the deep homology of TFE/MiT-regulated phagocytes with the functional convergence of coagulation effector cells that arose through lineage-specific recruitment. This architectural duality underscores how the immune system balances the preservation of conserved legacies with the flexibility of lineage-specific adaptations, fundamentally reshaping our understanding of immune assembly across Bilateria.

## Materials and methods

### Ethics and experimental animals

The experimental animals used in this study included healthy, disease-free adult individuals of standard commercial size across all examined species. Specifically, *L. vannamei*, *C. semilaevis*, and *C. gigas* were purchased from the aquaculture market in Qingdao, Shandong, China; *C. plagiosum* were purchased from the fish and aquarium market in Xiamen, Fujian, China; *L. morii* were purchased from Dandong, Liaoning, China; *C. intestinalis* were kindly obtained from Ocean University of China, and *C. hongkongensis* were kindly provided by the South China Sea Research Institute of the Chinese Academy of Fishery Sciences. All animals were temporarily maintained in a recirculating aquarium system equipped with an aeration pump. For blood and hemolymph collection, samples from oysters, shrimp, and tonguefish consisted of two biological replicates each, whereas those from the lamprey, shark, and tunicate consisted of three or more (≥3) biological replicates. Biological replicates and quality metrics for all species are summarized in Table S1. All aspects of animal transportation, breeding, and animal experimentation were approved by the Experimental Ethics Review Committee of the Yellow Sea Fisheries Research Institute of the Chinese Academy of Fishery Sciences (YSFRI-2023038).

### Sample collection and cell preparation

Hemolymph was extracted from the pericardial cavity (oyster) or the back (shrimp), or heart (tunicate) and put into an anticoagulant containing 0.1 mM EDTA and 3% sea salt on ice. Following centrifugation at 500 × g for 7 min at 4°C, the supernatant was removed, and the cell pellet was resuspended in PBS (Servicebio, #G0002). The above step was repeated twice. After the cell suspension was filtered with a 40 μm cell strainer (FALCON, #352340), the cells were counted with a hemocytometer (INCYTO) and diluted to obtain 1,000 cells per μL.

Blood was extracted from lampreys (*L. morii*), sharks (*C. plagiosum*) and tonguefish (*C. semilaevis*) and subsequently put into an anticoagulant with 0.1 mM EDTA. Density gradient centrifugation was used to remove some red blood cells to minimize the effect of excessive red blood cells. In details, 10 ml of 31% Purcell (Solarbio, #P8370) was added to a 50 ml centrifuge tube, followed by the slow addition of 10 ml of 54% Percoll below the previous layer. 15 ml of cell suspension was slowly added to the top layer and centrifuged (centrifuged at 4 °C and 400 × g for 10 min). After centrifugation, the target cell layer was collected from the 31% to 54% Percoll layer. After the cell suspension was filtered with a 40 μm cell strainer (FALCON, #352340), the cells were counted with a hemocytometer (INCYTO) and diluted to obtain 1,000 cells per μL.

### scRNA-seq library construction and sequencing

The DNBelab C Series high-throughput single-cell RNA library preparation set (MGI, #940-000519-00) was utilized for scRNA-seq library preparation. In brief, the single-cell suspensions were converted to barcoded scRNA-seq libraries through steps including droplet encapsulation, emulsion breakage, mRNA capture bead collection, reverse transcription, cDNA amplification and purification. The cDNA was then fragmented into short segments ranging from 300 to 500 bp. Indexed sequencing libraries were meticulously constructed following the manufacturer's protocol. Quality was assessed using Qubit ssDNA Assay Kit (Thermo Fisher Scientific) and Agilent Bioanalyzer 2,100. All libraries were further sequenced by the MGISEQ-2,000 or DNBSEQ-T1 sequencing platform with pair-end sequencing. The sequencing reads contained 30-bp read 1 (including the 10-bp cell barcode 1, 10-bp cell barcode 2 and 10-bp unique molecular identifiers (UMI)), 100-bp read 2 for gene sequences and 10-bp barcodes read for sample index.

### Single-cell RNA-seq data processing

Raw scRNA-seq reads were processed using the DNBelab_C_Series_HT_scRNA-analysis-software (V2.0.7), available at https://github.com/MGI-tech-bioinformatics/DNBelab_C_Series_HT_scRNA-analysis-software. For genomic alignment, reference genomes and their corresponding structural annotations for the shrimp, tunicate, shark, and tonguefish were obtained from the NCBI RefSeq database (assembly nos. GCF_003789085.1, GCF_000224145.3, GCF_004010195.1, and GCF_000523025.1, respectively). The reference genome for the oyster (*C. hongkongensis*) is currently unpublished and was kindly provided by the authors of a previous study (Meng et al. 2022). The full-length transcriptome reference for the lamprey was obtained from a recent publication (Huang et al. 2024) and downloaded from the Figshare repository (https://doi.org/10.6084/m9.figshare.26036284).

Standard downstream analyses for each library were primarily performed using Seurat v4 (Hao et al. 2021). To obtain high-quality gene reads, we first filtered out cells with fewer than 200 genes and 500 transcripts detected. Based on species-specific transcriptional characteristics, cells exhibiting excessive gene counts or high mitochondrial gene expression were also excluded (Table S1). Potential doublets in each library were tested using DoubletFinder (v2.0.3) (McGinnis et al. 2019), with an estimated doublet rate of 10%. Following quality control, each library was normalized using the *SCTransform* function with default parameters. For each species, individual libraries were integrated using the “SCT” method within the *FindIntegrationAnchors* framework. Dimensionality reduction was performed via Principal Component Analysis (PCA) using the top 30 principal components. The neighborhood graph was computed based on the PCA space, and cells were clustered using the *FindClusters* function (resolution = 0.5) and visualized via Uniform Manifold Approximation and Projection (UMAP). DEGs for each cluster were identified using the *FindAllMarkers* function (min.pct = 0.1, logfc.threshold = 0.5, test.use = “wilcox”). We annotated each cell type in vertebrates using marker genes that have been extensively cited in literature. For invertebrates, we used highly expressed immune-related genes for annotation (Tables S2 to S7).

### Gene functional annotation and enrichment

To assign functional attributes to the identified genes, we performed functional annotation against the NCBI Non-Redundant (NR) protein database. Furthermore, all protein sequences were mapped to the eggNOG (v5.0.2) (Huerta-Cepas et al. 2019) using eggNOG-mapper to retrieve corresponding GO terms. To facilitate downstream analysis, we constructed species-specific OrgDb packages using the *makeOrgPackage* function from the AnnotationForge (v1.40.1) R package. Finally, GO enrichment analysis and subsequent visualizations were performed using the clusterProfiler (v4.6.2) (Wu et al. 2021) package.

### SAMap analysis

In addition to the six datasets generated in our study, we incorporated public blood single-cell transcriptomes from turtles (http://www.bmkmanu.com/portfolio/demo), monkeys (https://www.10xgenomics.com/datasets/10-k-monkey-pbm-cs-multiplexed-2-cm-os-3-1-standard-6-0-0), and humans (https://www.10xgenomics.com/datasets/33-k-pbm-cs-from-a-healthy-donor-1-standard-1-1-0) to expand our comparative scope. To ensure a focused analysis of immune landscapes, we refined the datasets by excluding non-target populations: erythrocytes were removed from all vertebrate datasets, while non-immune cells—including muscle and epithelial cells in the oyster and tunicate—were also excluded. To mitigate sequencing noise and enhance data robustness, every 10 cells of the same type within each species were aggregated into pseudo-meta cells.

Multi-species comparisons were performed using the Python package SAMap (v1.0.15) (Tarashansky et al. 2021). This method enables the mapping of single-cell transcriptomes between distantly related species while maintaining robustness in accounting for technical batch effects across different platforms. To ensure accurate homology identification, conserved orthologous groups between all species pairs were calculated using OrthoFinder (v2.3.7) (Emms and Kelly 2019) to construct a comprehensive gene-gene bipartite graph. The processed Seurat objects were converted to.h5ad format via the R package SeuratDisk (v0.0.9019) and loaded into Python (v3.7.16).

We first integrated the six vertebrate datasets by executing pairwise SAMap alignments with the *sm.run* function. The resulting integrated manifold was visualized via UMAP-based dimensionality reduction using the *sc.pl.umap* function in Scanpy (v1.8.2) to generate a cross-species projection of cell-type clusters. To evaluate global conservation across all nine species, transcriptomic similarity scores between cell types were quantified using the *get_mapping_scores* function. These scores were subsequently visualized as a global similarity matrix using the pheatmap (v1.0.12) R package. Finally, to identify the molecular drivers of these cellular correspondences, gene pairs contributing to cell-type homology were extracted using *GenePairFinder* with a significance threshold of 0.05.

### Pairwise cross-species cell similarity inference and computation of shared highly expressed homologous genes

To systematically evaluate transcriptional similarity across species, we integrated the computation of orthologous gene expression profiles with Kullback-Leibler divergence (KLD) analysis, following established comparative frameworks (Levy et al. 2021; Nagahata et al. 2022). Our analytical workflow comprised the following key steps:

First, we identified 4,455 conserved orthologous groups (OGs) across all nine species using OrthoFinder with default parameters. Genes not assigned to these core OGs were excluded to ensure high-confidence homologous comparisons. For OGs containing multiple paralogs within a given species, raw expression values were summed to generate composite “ortho-gene” profiles, resulting in a standardized matrix of 4,455 ortho-genes for each species.
To provide a standardized foundation for cross-species comparisons, we transformed these ortho-gene profiles into species-specific Fold Change (FC) matrices, thereby capturing cell-type-specific signatures while mitigating inter-species baseline variations. For each of the nine species, we calculated the geometric mean expression of ortho-genes per cell type. To account for variations in sequencing depth, these values were normalized by the median cell size. We then introduced a regularization factor (pseudocount = 0.05) to determine a robust FC relative to the global median expression of all cell types within that species. These individual FC matrices were subsequently integrated into joint orthologous expression matrices for all possible species pairs, serving as the direct input for downstream similarity computations.
To quantify cross-species cell-type similarities, we calculated metrics based on the KLD between cell-type pairs using the joint FC matrices. Since lower KLD values indicate higher transcriptomic similarity, we mathematically inverted these distances into similarity scores to facilitate comparison. We then applied a threshold based on the 88th percentile of these similarity scores (representing the top 12% of most similar connections) to identify significant clusters, which were visualized as linking ribbons in the Circos plot. Finally, for the cell-type groups connected by these links, we identified shared orthologous markers, defined as those maintaining a consistent FC > 1.1 in both species, with statistical significance assessed using a paired Wilcoxon test between ingroup and outgroup populations.

### Ancestral state reconstruction of phagocytic-active genes

To reconstruct the evolutionary trajectory of phagocytic cell-specific gene expression, we performed ancestral state inference based on the 4,455 conserved OGs. A species-by-OG binary matrix was constructed by binarizing the species-specific FC matrices described above. For each species, we focused on the phagocytic cell clusters identified via transcriptomic similarity (Fig. 3a). An OG was assigned a state of “1” (active) if its FC exceeded 1.1 in any of the constituent phagocytic subclusters; otherwise, it was assigned a state of “0” (inactive).

Ancestral states for internal nodes were then inferred using maximum parsimony via the asr_max_parsimony function from the castor (v1.8.3) R package (Louca and Doebeli 2018), with the species phylogenetic tree imported using ape (v5.8) (Paradis et al. 2004). This method identifies the most likely evolutionary path by minimizing state transitions across the phylogeny. Gene “gains” (0 to 1) and “losses” (1 to 0) were quantified across branches and normalized by branch length (changes per million years, Myr) to account for temporal variation. Evolutionary transitions and ancestral states were visualized using the phytools (v2.3.0) package (Revell 2012).

### Phylogenetic reconstruction of MiT/TFE and C/EBP families

To investigate the evolutionary relationships and conservation of key TFs, we performed comprehensive phylogenetic analyses for the MiT/TFE and C/EBP families. For the MiT/TFE family, protein sequences from the nine study species were supplemented with homologous sequences retrieved from the NCBI database, including *Danio rerio* (zebrafish), *Drosophila melanogaster* (fruit fly), *Branchiostoma lanceolatum* (lancelet), and *Crassostrea gigas* (Pacific oyster). To clarify the subfamily topology and ensure robust rooting, sequences from the broader bHLH-ZIP superfamily, specifically the MYC and USF subfamilies, were also obtained from NCBI and included as outgroups. Similarly, for the C/EBP family, sequences from the nine study species were integrated with NCBI-sourced sequences from *Danio rerio*, *Electrophorus electricus* (electric eel), *Lepisosteus oculatus* (spotted gar), *Branchiostoma lanceolatum*, *Petromyzon marinus* (sea lamprey), and *Crassostrea gigas*.

Multiple sequence alignments (MSA) were conducted using MAFFT (v7.520) with the L-INS-i iterative refinement strategy, which is optimized for high-accuracy alignment of sequences containing conserved domains (Katoh and Standley 2013). To improve the signal-to-noise ratio in the phylogenetic inference, raw alignments were trimmed using ClipKit (v1.3.0) in default mode to remove poorly aligned or phylogenetically uninformative sites. Maximum Likelihood (ML) phylogenetic trees were subsequently reconstructed using IQ-TREE 3 (Minh et al. 2020; Steenwyk et al. 2020). The best-fit amino acid substitution model was automatically determined by the built-in ModelFinder based on the Bayesian Information Criterion (BIC) (Kalyaanamoorthy et al. 2017). Branch support was evaluated using 1,000 ultrafast bootstrap replicates to ensure the reliability of the inferred nodes. The resulting phylogenetic trees in Newick format were visualized and annotated using the Interactive Tree Of Life (iTOL, v7) web server. Final adjustments to tree aesthetics, including branch scaling and node labeling, were performed within the iTOL environment to highlight the evolutionary divergence across the studied lineages.

### hdWGCNA analysis

We employed the standard hdWGCNA (v0.4.5) workflow to construct gene co-expression networks across species (Langfelder and Horvath 2008; Morabito et al. 2023). First, the *SetupForWGCNA* function was used to store the data in a Seurat object, with the input gene set comprising all genes from 4,455 conserved orthogroups shared among the species. Next, metacells were generated using the *MetacellsByGroups* function (k = 25, max_shared = 10). All subsequent module construction steps were performed using default parameters. To visualize the distribution of co-expressed gene modules, we applied the *RunModuleUMAP* function (n_hubs = 20, n_neighbors = 15, min_dist = 0.1). Finally, specific phagocytosis-related genes were extracted and visualized as a network using the ggraph (v2.2.1) R package (Pedersen 2020).

### Virtual gene knockout analysis

To investigate the regulatory influence of the MiT/TFE family on the ancestral immune program, we performed virtual gene knockout analysis using scTenifoldKnk (v1.0.3). For each species, the gene expression matrix was extracted specifically from the phagocytic cell subclusters that exhibited transcriptomic similarity (as identified in Fig. 3a). The analysis focused on a subset of 1,131 ancestral phagocytic-active genes, which were determined through ancestral state reconstruction (ASR) as described above.

Virtual knockouts were executed by systematically silencing specific members of the MiT/TFE family within each species-specific regulatory manifold. The gene regulatory network construction and comparison were performed using the scTenifoldKnk function with the following parameters: quality control was disabled (qc = FALSE), the number of individual networks was set to 10 (nc_nNet = 10), and the number of cells sampled per network was 500 (nc_nCells = 500). Genes significantly affected by the VKO (differentially regulated genes, DRGs) were identified using a significance threshold of adjusted *P*-value (padj) < 0.05. To further elucidate the biological impact of these perturbations, GO enrichment analysis was conducted on the resulting DRGs to identify conserved functional modules regulated by the MiT/TFE family.

### Activation of invertebrate hemolymph clotting

Hemolymph was collected from oysters, shrimp, and tunicate and immediately added to 1 ml of anticoagulant with 10% sodium citrate (Solarbio, #YS175647) and 3% sea salt on ice. After the cell suspension was filtered with 40 µm cell strainers, the supernatant was removed by centrifugation (4 °C at 400 × g for 15 min), and the pellet was resuspended in 1 ml of anticoagulant with 3% sea salt. Blood coagulation was initiated by adding 100 µl of 1 M CaCl_2_, followed by gentle mixing and incubation at room temperature for 10 min (see (Chaikeeratisak et al. 2012)). The cell suspensions (300 µl) were attached to polysine microscope adhesion slides (Solarbio, #YA0170) by cell smears. Cell slides were then dried at 37°C for 15 min in drying oven.

### Fluorescence in situ hybridization

Cell slides were fixed using in situ hybridization fixative (Servicebio, #CR2207020) for 20 min and washed in PBS (pH 7.4) on a decolorization shaker three times for 5 min each. Proteinase K (Servicebio, #G1234) (20 μg/ml) was added at 37 °C for digestion. After being washed with pure water, PBS was used to wash cell slides 3 times, 5 min each time. The pre-hybridization solution (Servicebio, China) was then added, and cell slides were incubated at 37 °C for 1 h. After dumping the pre-hybridization solution, target gene probe-containing hybridization solution (Servicebio, China) was dropped. Probes information was shown in Table S18. Cell slides were kept in the incubator overnight. To wash the hybridization solution, cell slides were rinsed with 2 × SSC (Servicebio, China), 37 °C for 10 min, 1 × SSC, 37 °C for 2 × 5 min, 0.5 × SSC at room temperature for 10 min. Cell slices were added with 60 μL preheated corresponding branch probe hybridization solution in a wet box at 40 °C for 45 min. In this process, 50 ml 2 × SSC was added to the bottom of the wet box to prevent dry slices. After pouring out hybridization solution, and the cell slices were rinsed with 2 × SSC, 1 × SSC, 0.5 × SSC, 0.1 × SSC preheated at 40 °C for 5 min at 40 °C. The hybridization solution containing the signal probe was added and cell slices were incubated at a dilution ratio of 1: 400.42 °C for 3 h. After being washed by the following SSC: 2 × SSC, 37 °C for 10 min, 1 × SSC, 37 °C for 2 × 5 min, 0.5 × SSC 37 °C for 10 min, DAPI staining solution (Servicebio, China) was added to the cell slices in the dark condition for 8 min. The anti-fluorescence quenching sealing agent was added to the cell slices after washing. Sections were observed under a microscope slide scanner (Pannoramic MIDI), and images were collected. (UV excitation wavelength 330-380 nm, emission wavelength 420 nm, blue light; CY3 red light excitation wavelength of 510-560, emission wavelength of 590 nm, red light.)

### Stereo-cell technology chip preparation and sequencing

The HM suspension prepared from the oyster (Pacific oyster, *Crassostrea gigas*) and shrimp (*L. vannamei*) was used here. A 20 μL sample of cell suspension was meticulously deposited onto pre-prepared Stereo-cell chips, then evenly distributed using a pipette tip. The chips were left to stand at room temperature for 10 minutes to facilitate cell adhesion and capture. For cell fixation, the chips were immersed in pre-chilled methanol for 30 minutes at −20°C. Subsequently, DAPI staining solution was applied, and the chips were kept in darkness for 5 minutes. Following this, the chips underwent two washes with 0.1×SSC Buffer (Thermo, #AM9770), then were coated with glycerol (Sangon Biotech, #4100854050), and sealed with a coverslip for subsequent imaging. Imaging was performed using a Motic Custom PA53 FS6 microscope. Cells were permeabilized on the chips using 0.1% pepsin (Sigma, #P7000) for 30 seconds, followed by a wash with 0.1 × SSC Buffer. Next, 200 μL of pre-hybridization solution was added to the chip, which was then incubated for 5 minutes at 55°C and washed again with 0.1 × SSC Buffer. Cell-derived transcripts were reverse transcribed for 3 hours at 42°C using Golden Reverse Transcriptase Mix (GCATBio, #LS-EZ-E-00024O). The chips were then treated with cDNA Release buffer (STOmics, #1000028512) for approximately 10 hours at 55°C and subsequently washed with nuclease-free water. The cDNA was purified using 0.7 × VAHTS DNA Clean Beads (Vazyme, #N411-03), resuspended in 200 μL of amplification mix, and PCR amplified with the following parameters: an initial denaturation at 95 °C for 5 minutes, followed by 12 cycles of denaturation at 98 °C for 20 seconds, annealing at 58 °C for 20 seconds, extension at 72 °C for 3 minutes, and a final extension at 72 °C for 5 minutes. The amplified cDNA was purified once more and subjected to sequencing on the MGI DNBSEQ T1 sequencer at the China National Genebank.

### Stereo-cell data processing

To obtain the spatial gene expression matrices, we matched data and assigned cells to DNBs by segmenting objects from DAPI images and aligning these with gene expression images. We began by aligning DAPI images with gene expression coordinates and converted gene expression data into 2D images using color-coded molecular identifiers for each DNB. DAPI images were pre-processed to remove noise and enhance contrast. Using the Fiji TrakEM2 plugin (Cardona et al. 2012; Schindelin et al. 2012), we manually identified homologous anatomical features between the tissue images and gene maps to calculate the affine transformation matrix.

Cell segmentation was conducted using CellProfiler pipelines (McQuin et al. 2018). Briefly, nuclei were identified from the ssDNA images using the Global Otsu thresholding method, and cell boundaries were defined via the Mutex Watershed algorithm, with diameters set between 10 and 40 pixels. The resulting cell masks were then transformed back to the gene expression coordinate system using the previously determined affine parameters. Finally, we employed the apply_cells module of the GEM_toolkit (https://github.com/BGI-Qingdao/GEM3D_toolkit/blob/dev/GEM_toolkit.py) to aggregate RNA transcripts into individual cells based on their spatial overlap with the defined cell boundaries. This process effectively converted the DNB-level coordinate data into a Cell Bin (CB) matrix, enabling single-cell resolution analysis while preserving spatial context.

The resulting CB matrices were imported into Seurat (v4.0) for downstream analysis. Quality control was performed by filtering out low-quality cells containing fewer than 50 detected genes. The data matrices were then standardized using the *SCTransform* method. PCA analysis was conducted with default settings, and the top 20 principal components were extracted for unsupervised clustering. Cell populations were identified using the *FindClusters* function with a resolution of 0.5. For high-dimensional data visualization, we employed UMAP. Finally, the identified cell clusters were projected back onto the original spatial coordinates of the Stereo-cell chip to reconstruct the spatial architecture of the circulating immune system across the studied species.

## Supplementary Material

msag109_Supplementary_Data

## Data Availability

The scRNA-seq data generated in this study have been deposited in the Genome Sequence Archive in National Genomics Data Center (Chen et al. 2021), China National Center for Bioinformation/Beijing Institute of Genomics, Chinese Academy of Sciences (GSA: CRA022255) that are publicly accessible at https://ngdc.cncb.ac.cn/gsa. The processed scRNA-seq data for each species can be accessed through the Figshare website (https://figshare.com/articles/dataset/Rds_files/28008557).
