# Supplementary material for: Evolutionary conservation and divergence of phagocytic and coagulation programs across bilaterian circulating immune cells
Source: Mol Biol Evol. 2026 Apr 27;43(5):msag109. doi: 10.1093/molbev/msag109 (PMC13186322; doi:10.1093/molbev/msag109)
Supplement: msag109_Supplementary_Data [file msag109_supplementary_data.zip › msag109_Supplementary_Data/Supplementary Material.docx]

Supplementary Material

**Evolutionary conservation and divergence of phagocytic and coagulation programs across bilaterian circulating immune cells**

**This supplementary material includes:**

Figures S1-S21

Legends for Tables S1-S18

**Other Supplementary Materials for this manuscript include the following:**

Tables S1 to S18


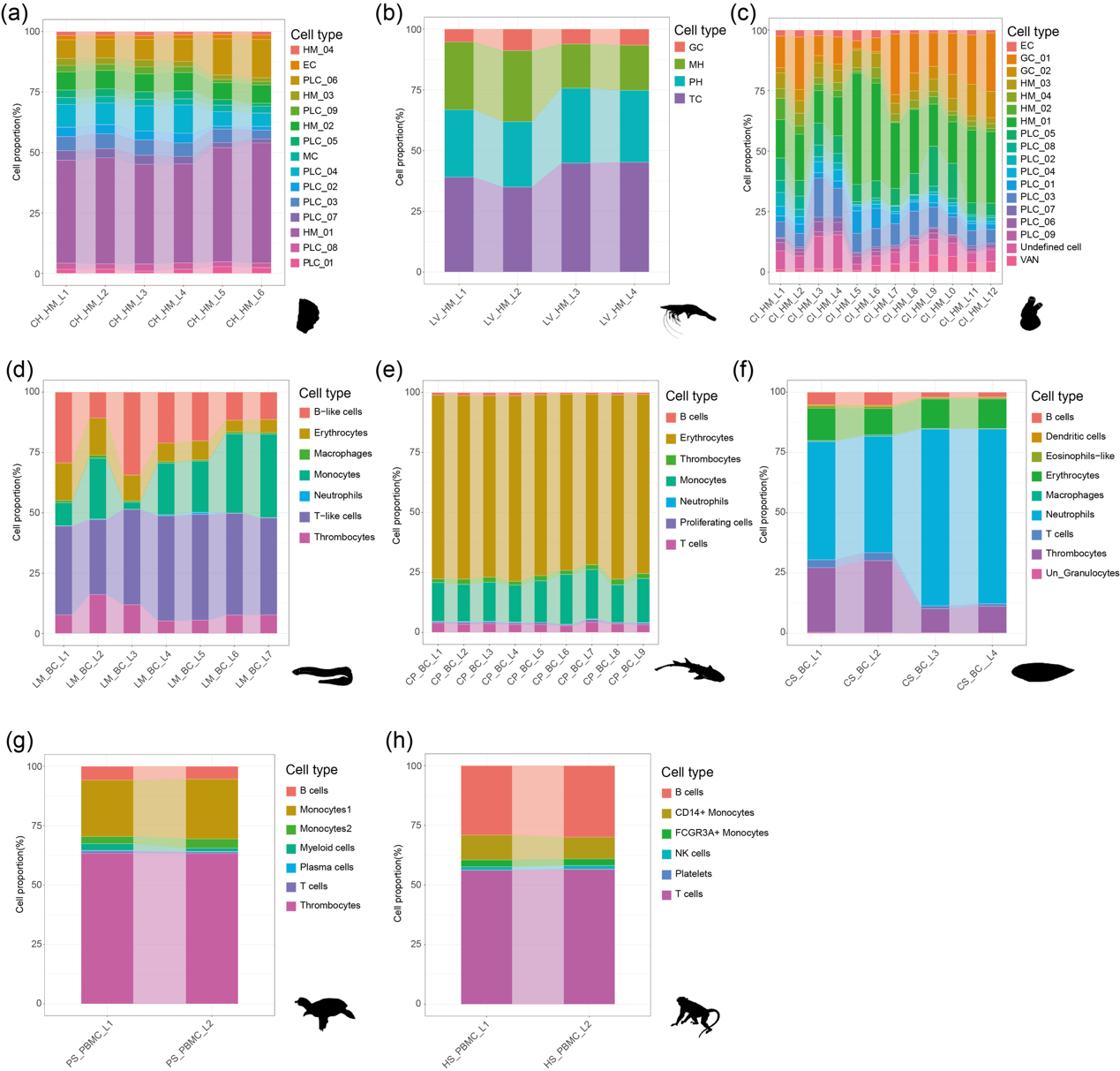


**Figure S1. The proportion of cell types in each library for each species.** (a-h) The cell proportions of individual libraries from the scRNA-seq datasets of oyster (a), shrimp (b), tunicate (c), lamprey (d), shark (e), tonguefish (f), turtle (g) and monkey (h).


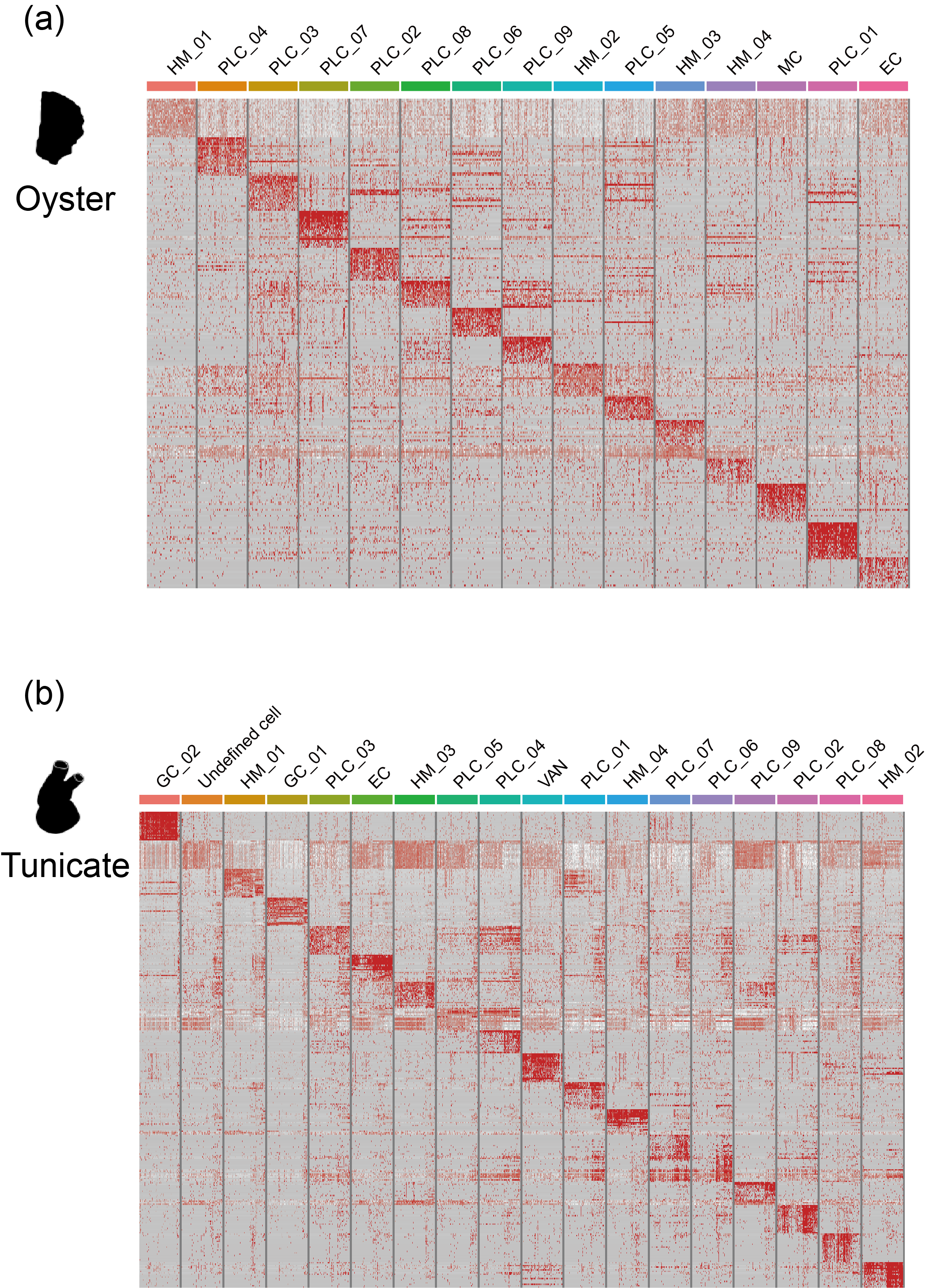


**Figure S2. Differential gene expression in hemocytes of oysters and tunicates.** (a-b) Heatmap showing the expression of the top 20 differentially expressed genes (DEGs) for each cell type in oyster (a) and tunicate (b).


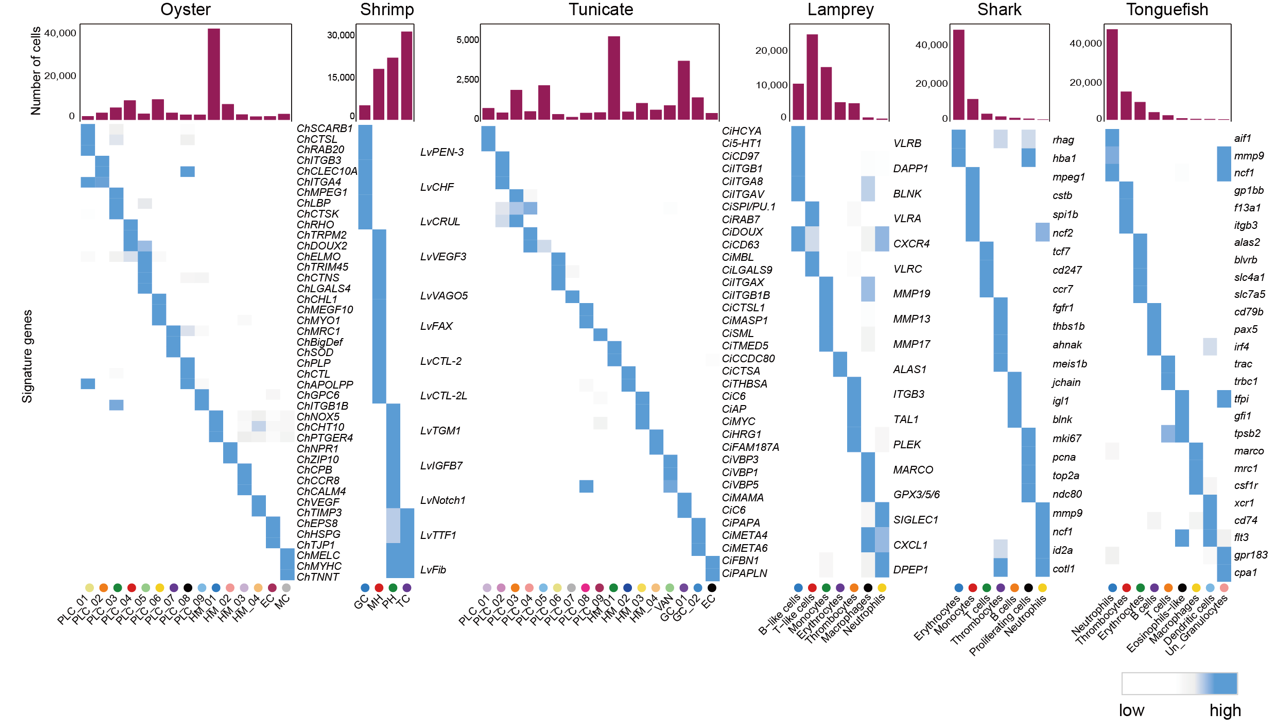


**Figure S3. Heatmap showing the expression of selected markers to identify cell types in oyster, tunicate, shrimp, lamprey, shark, and tonguefish.**


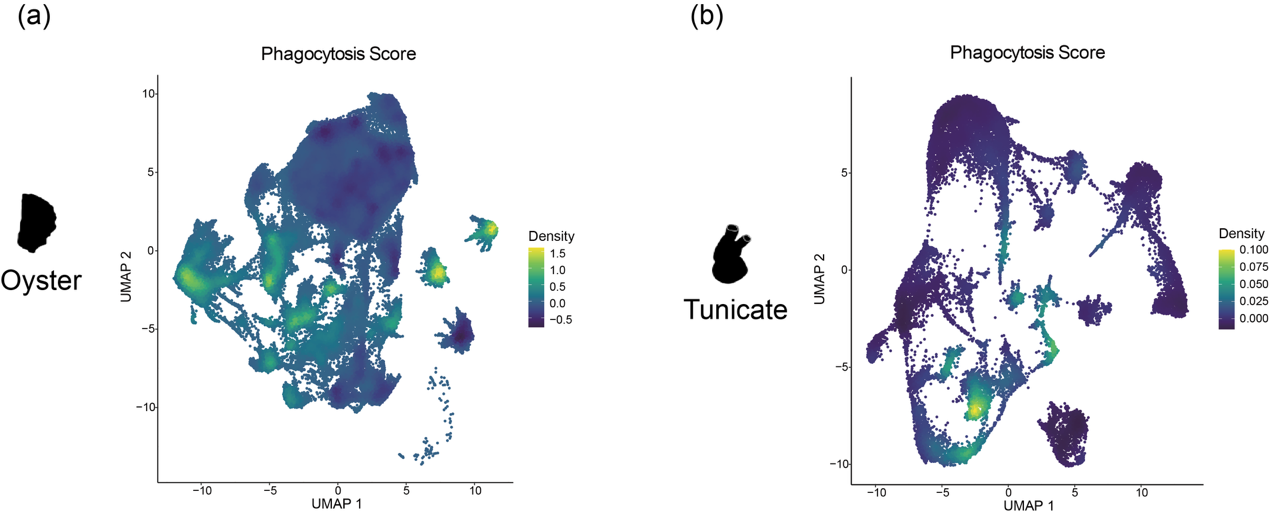


**Figure S4. The density plot displays expression patterns of phagocytosis-related genes in oyster (a) and tunicate (b).**


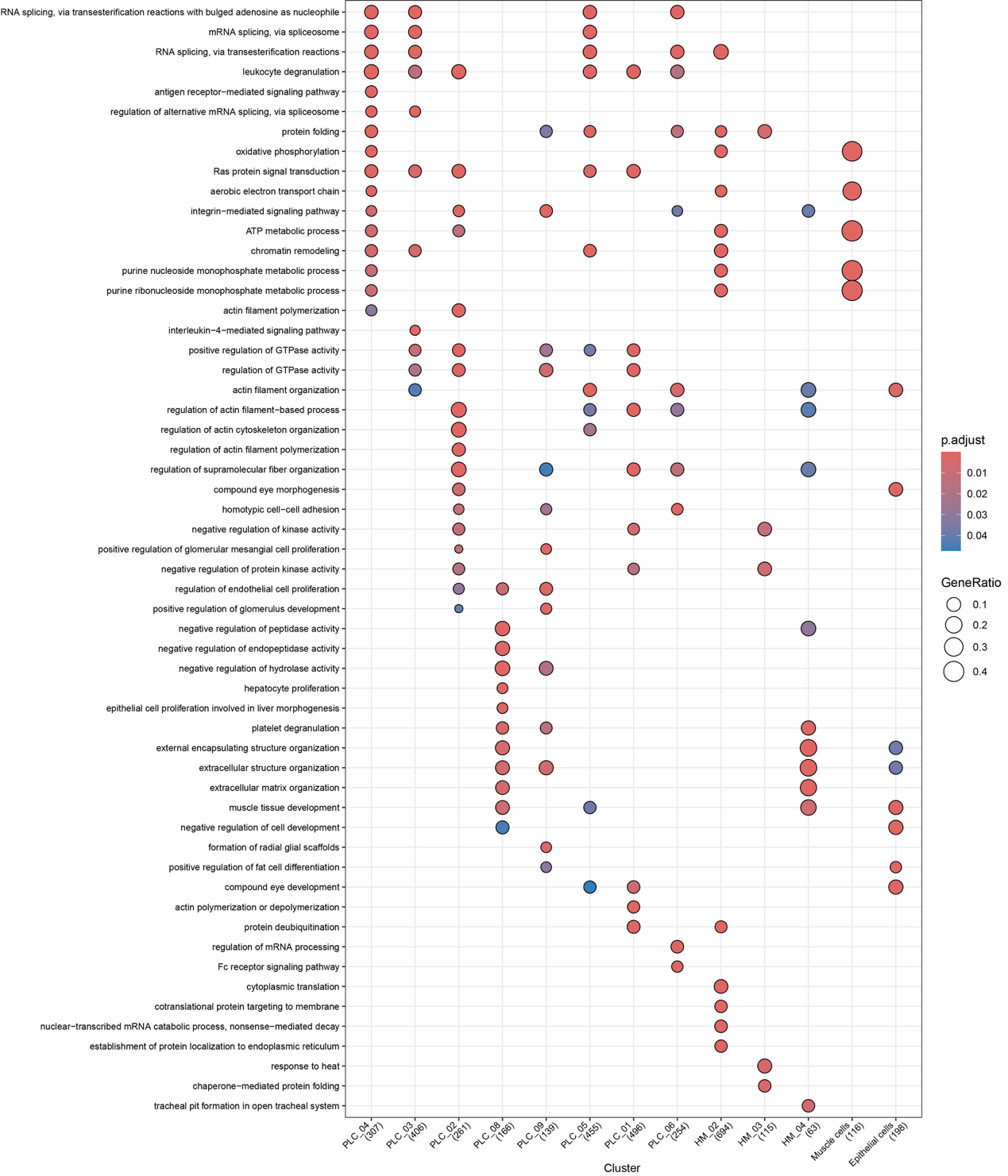


**Figure S5.** **Dot plot showing the functional enrichment of cluster-specific DEGs in oyster hemocytes.**


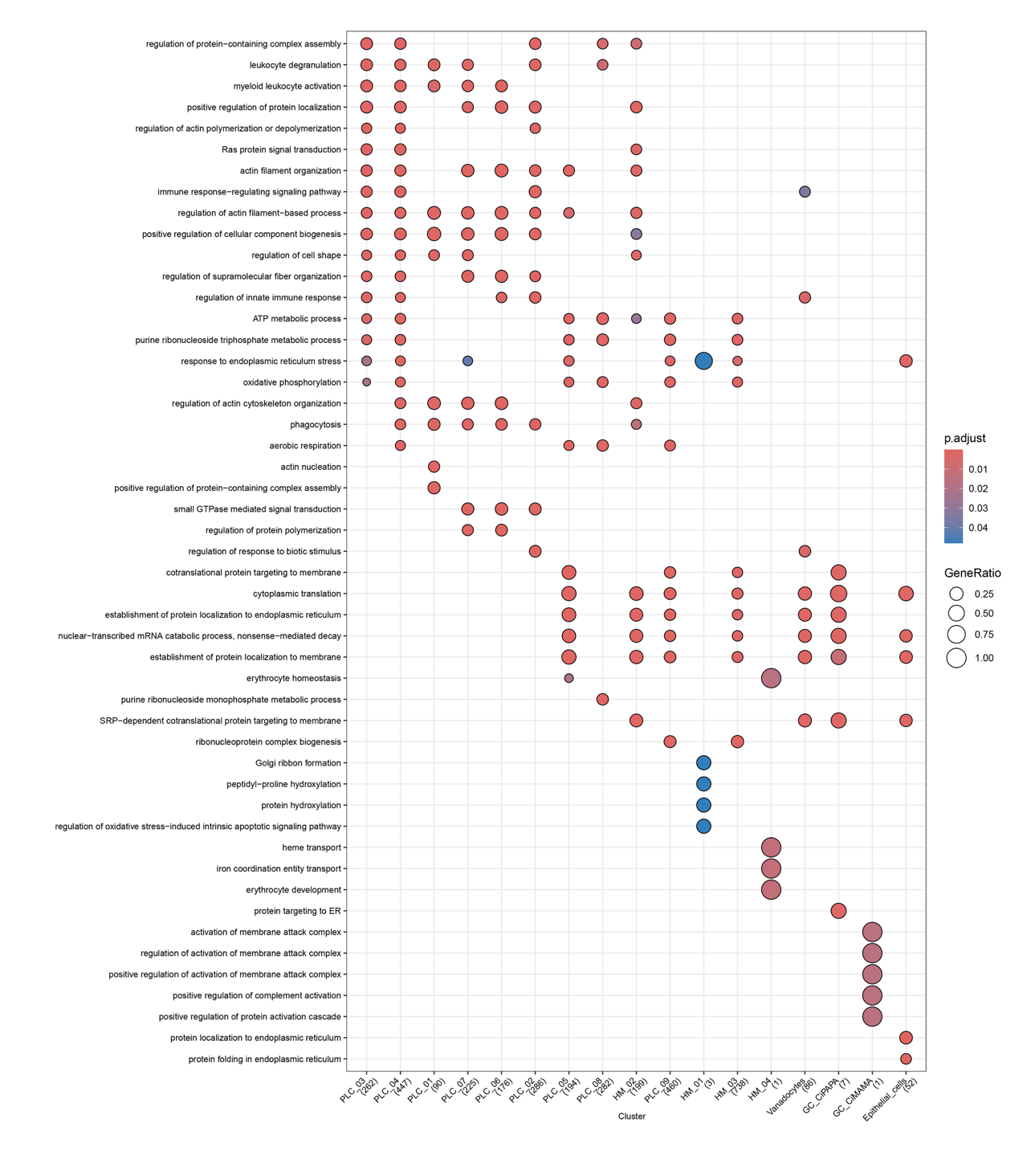


**Figure S6.** **Dot plot showing the functional enrichment of cluster-specific DEGs in tunicate hemocytes.**


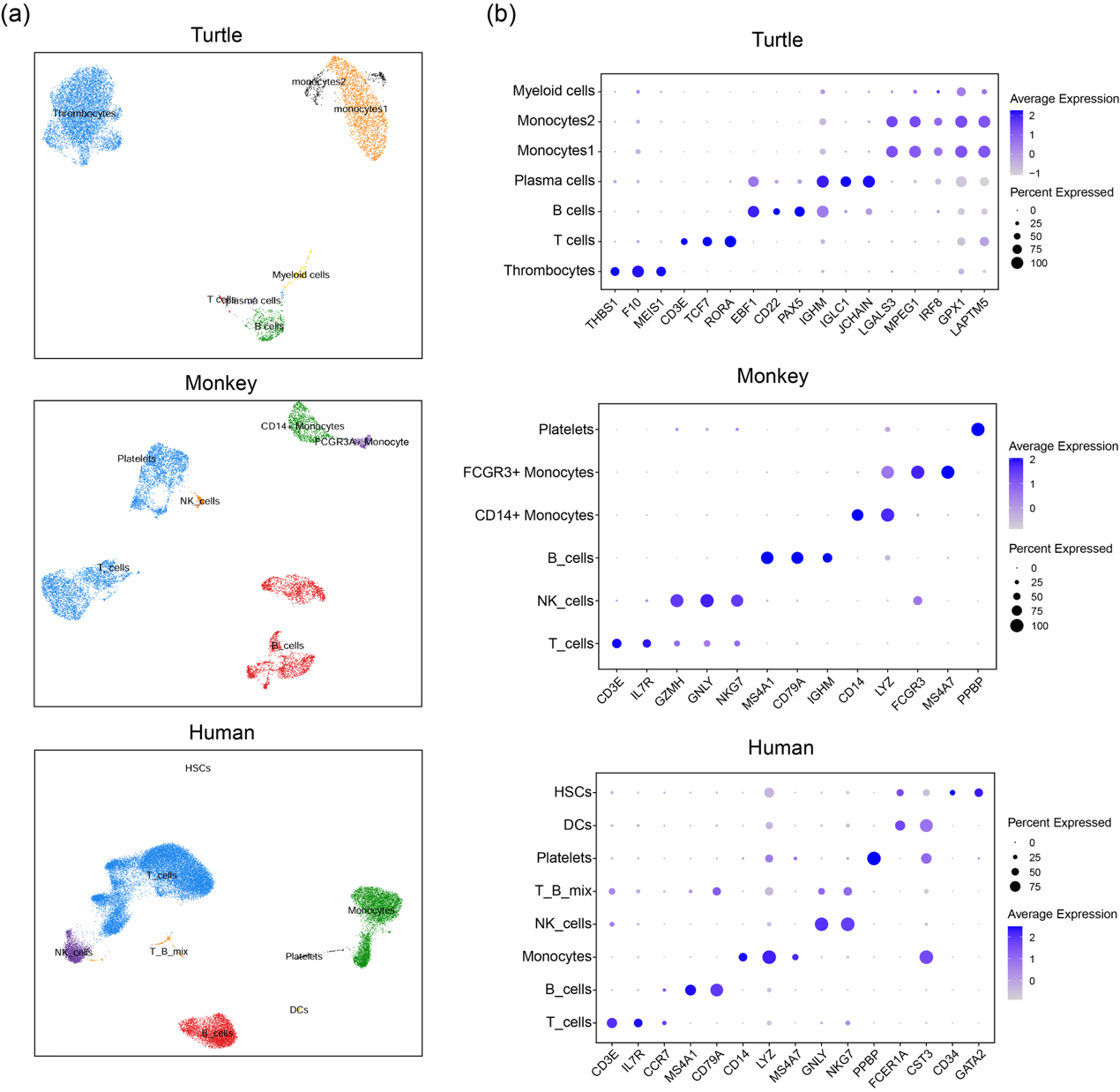


**Figure S7. Additional cell annotation information.** (a) UMAP visualization shows cell type annotation of turtle, monkey, and human. (b) DotPlot showing the expression of selected markers to identify cell types in turtle, monkey, and human.


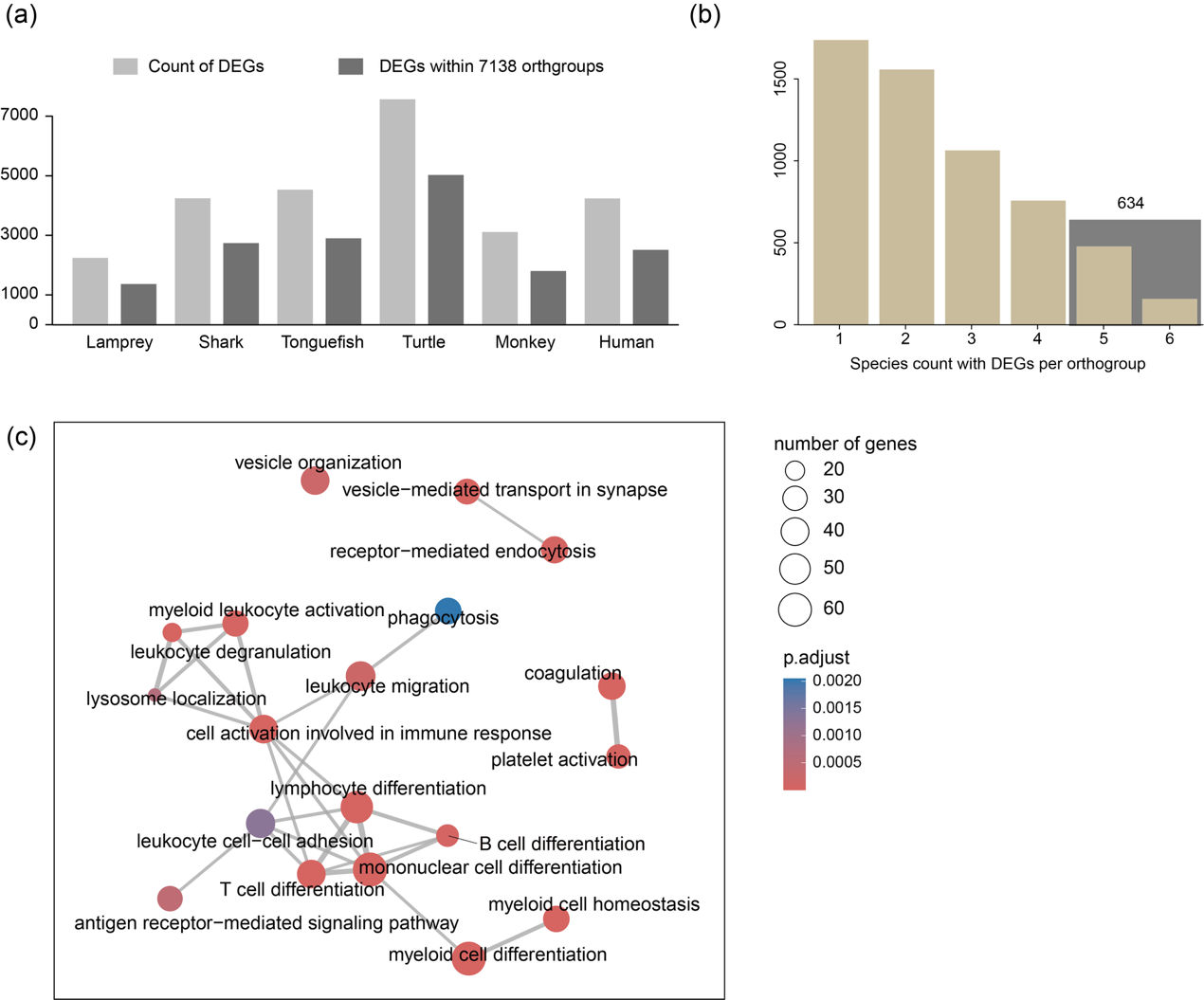


**Figure S8. Conservation and functional enrichment of vertebrate immune cell DEGs.** (a) Counts of DEGs in immune cells and their assignment to putative orthogroups. (b) Bar plot detailing the distribution of orthogroups containing immune cell DEGs shared across one to six vertebrate species. The x-axis indicates the number of species sharing these DEGs per orthogroup, and the y-axis represents the total count of such orthogroups. (c) GO functional enrichment analysis of the conserved DEGs in vertebrate immune cells.


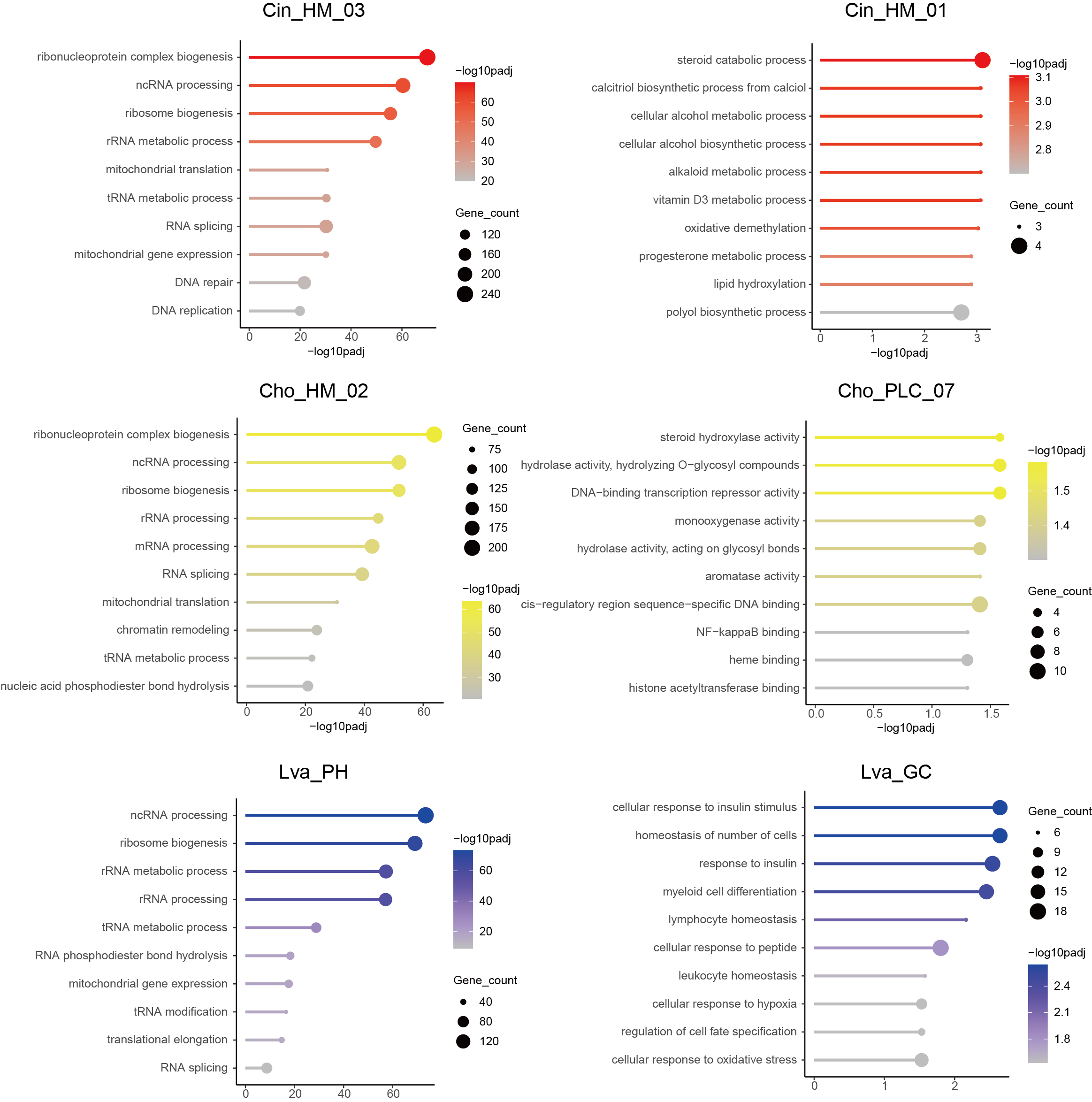


**Figure S9. Homologous gene characteristics shared across species.** GO term enrichment analysis of shared genes between specific hemocyte populations in oyster, shrimp, and tunicate with their analogous cell populations in vertebrates.


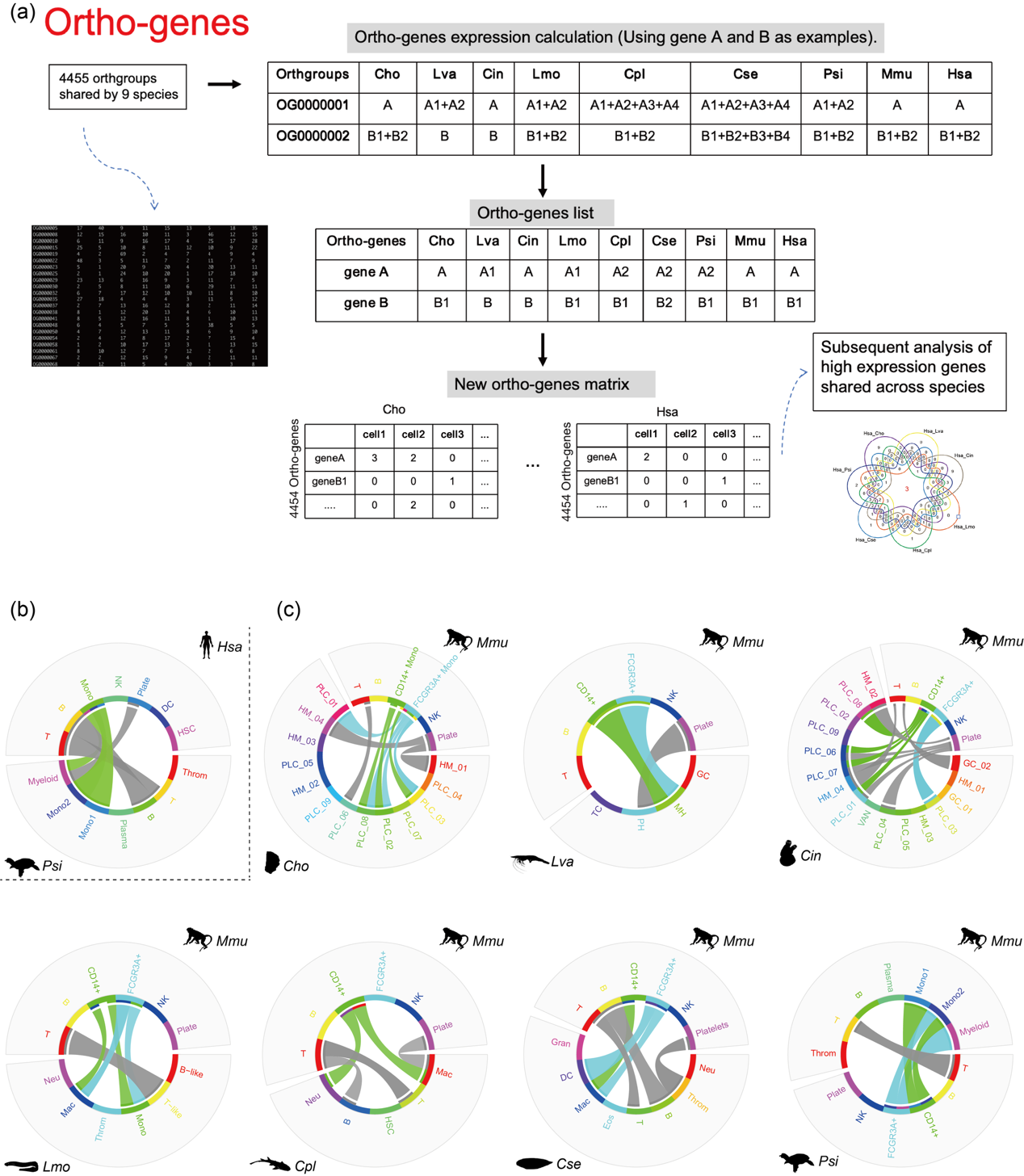


**Figure S10. Cross-species comparative analysis based on conserved orthogroups.** (a) The cross-species comparison workflow utilizing ortho-genes was implemented through the following steps: First, OrthoFinder was employed to identify 4,455 conserved orthogroups across the nine studied species. For each orthogroup within each species, we calculated the summed expression values when multiple genes were present (>1 gene per orthogroup) and designated the highest-expressed gene's identifier as the representative ortho-gene. This process generated a unified expression matrix encompassing all 4,455 ortho-genes across the examined species. Finally, this standardized matrix served as the foundation for subsequent screening of evolutionarily conserved genes. (b) The transcriptional similarity between human and turtle blood cells. Connections with Kullback-Leibler divergence (KLD) values above 88% are shown as arches linking pairs of cell types. (c) The circos plot displays transcriptional similarities between cell types from each species compared to monkey. Connections with KLD values above 88% are shown as arches linking pairs of cell types.


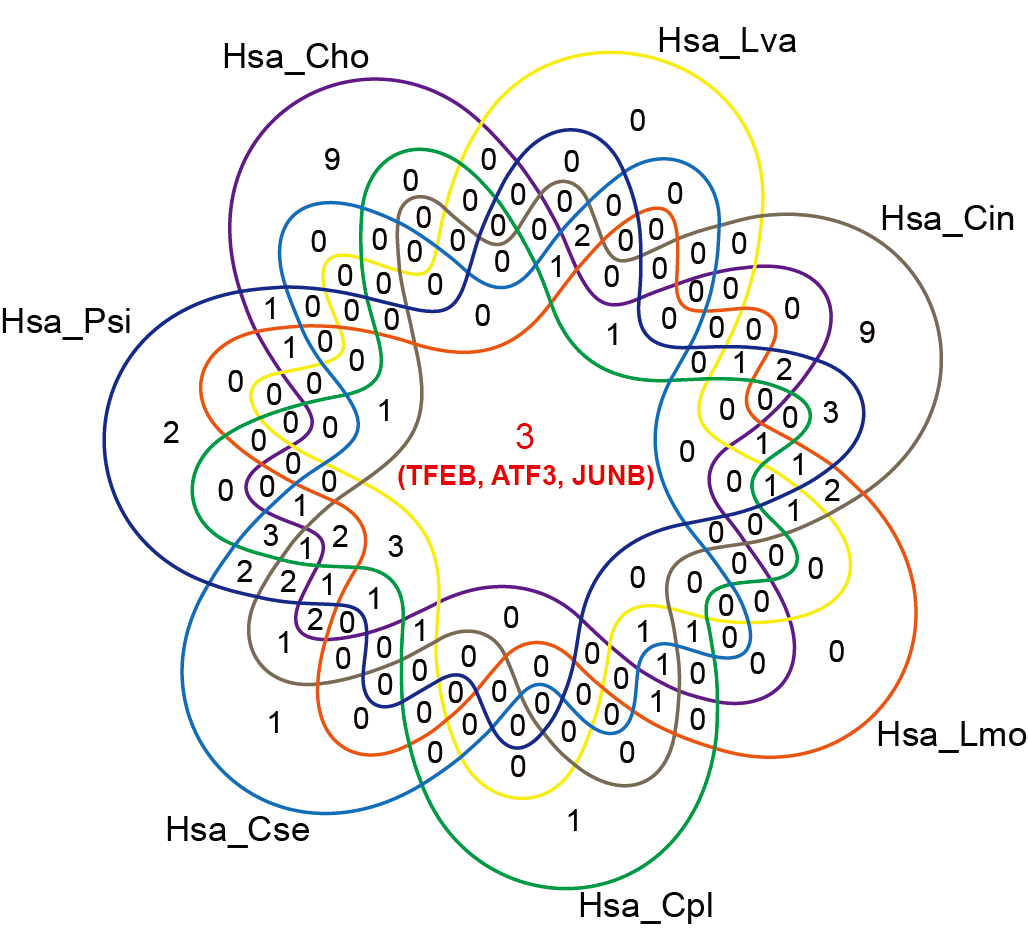


**Figure S11. Venn diagram shows the overlap of shared TFs between phagocytic cells in seven species and human.**


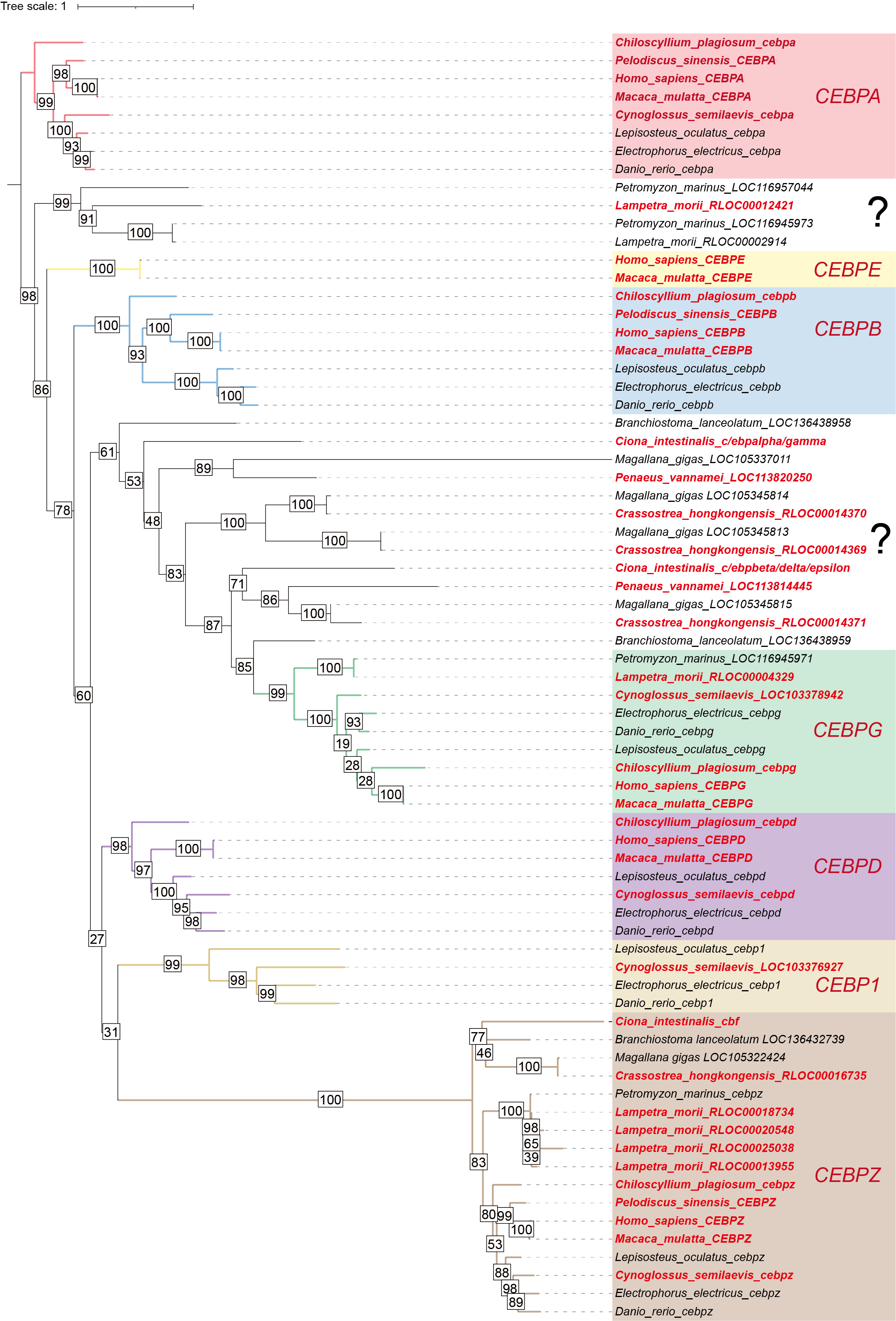


**Figure S12. Phylogenetic tree of the C/EBP family across species.** Branch colors indicate distinct orthologous clades within the C/EBP family. Sequences from species analyzed in this study are highlighted in bold red. Question marks indicate sequences that could not be clearly classified into specific subfamilies.


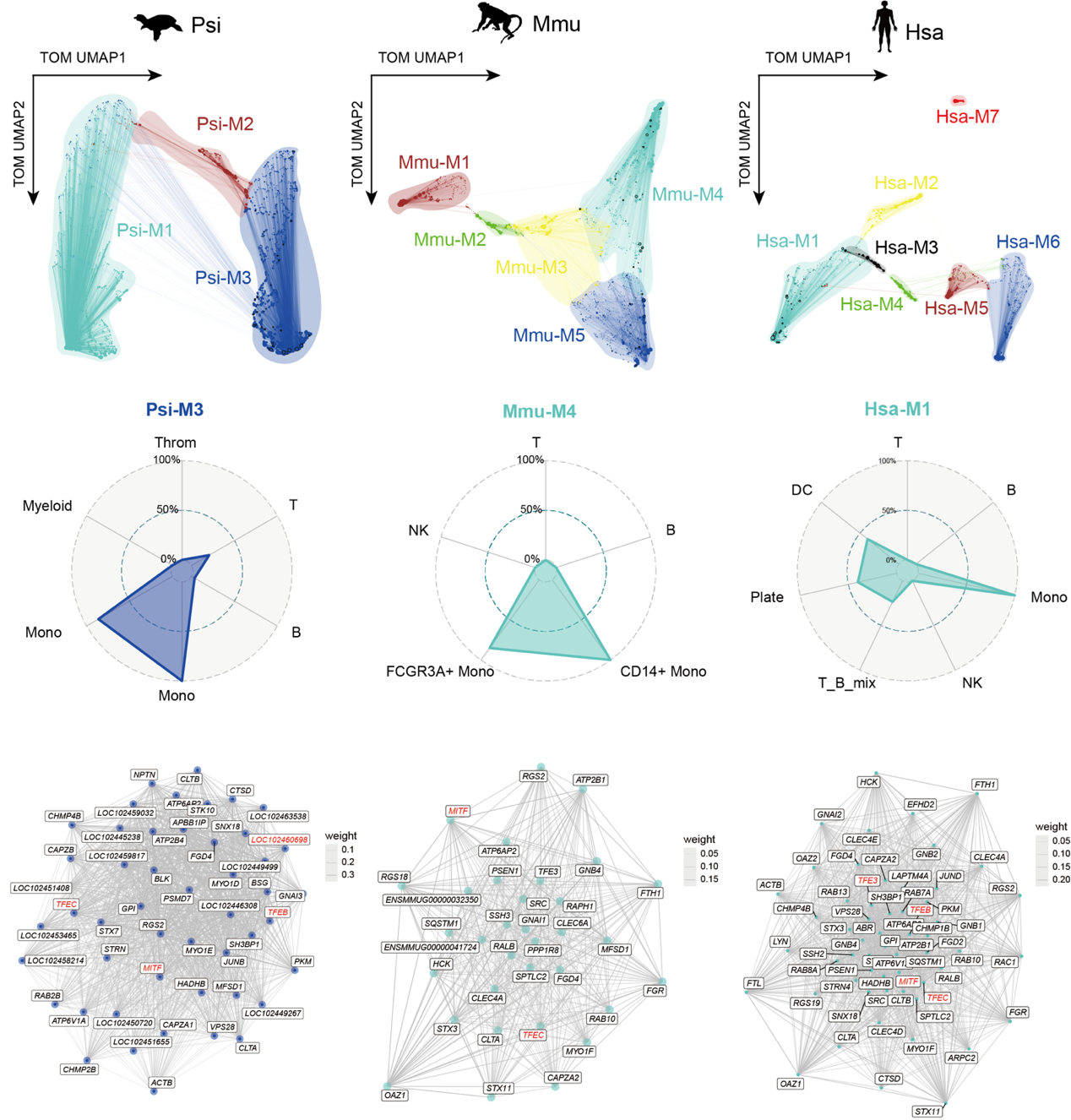


**Figure S13. Homologous transcriptional regulation of phagocyte evolution.** TOP: UMAP Plot shows the gene co-expression modules identified by hdWGCNA analysis based on 4455 orthgroups from turtle, monkey, and human. Nodes represent individual genes, with edges depicting significant co-expression relationships between genes and module hub genes. Node size scales with intramodular connectivity (kME values), while node coloration corresponds to module assignment. Middle: Radar plots visualizing the relative expression levels of selected modules across cell clusters in each species, with radial axes representing distinct clusters. Bottom: Network plot showing the co-expression relationships among 53 shared orthgroups (see fig. 3b) present in phagocyte-specific modules across species.


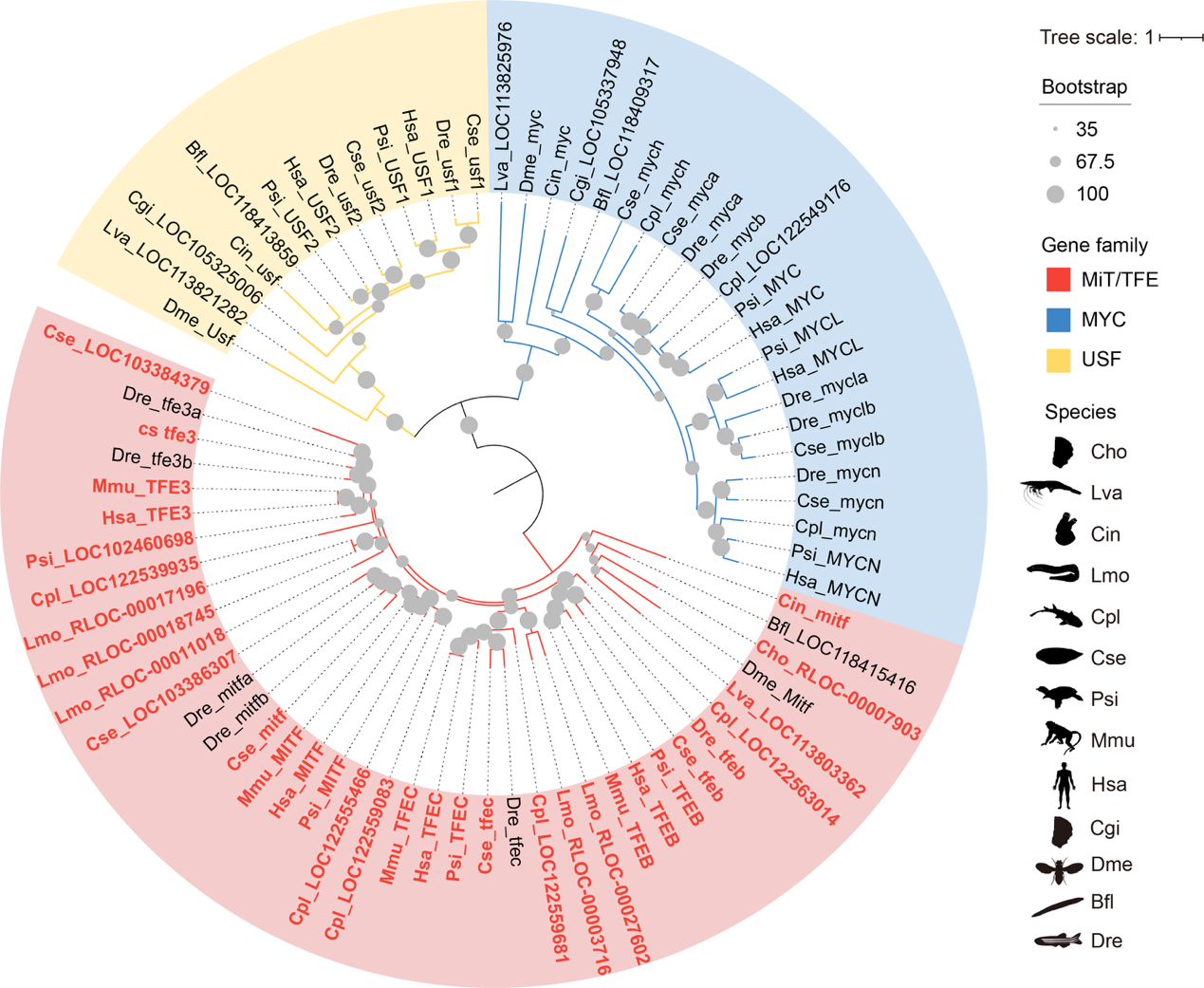


**Figure S14. Phylogenetic reconstruction of the MiT/TFE family and related bHLH-LZ subfamilies.** The circular tree delineates the evolutionary relationships among the MiT/TFE (red shading), MYC (blue shading), and USF (yellow shading) gene families across representative metazoan species. Nodal support is indicated by grey circles, with sizes proportional to their bootstrap values. Tip labels of MiT/TFE orthologs derived from the focal species investigated in this study are highlighted in red text.


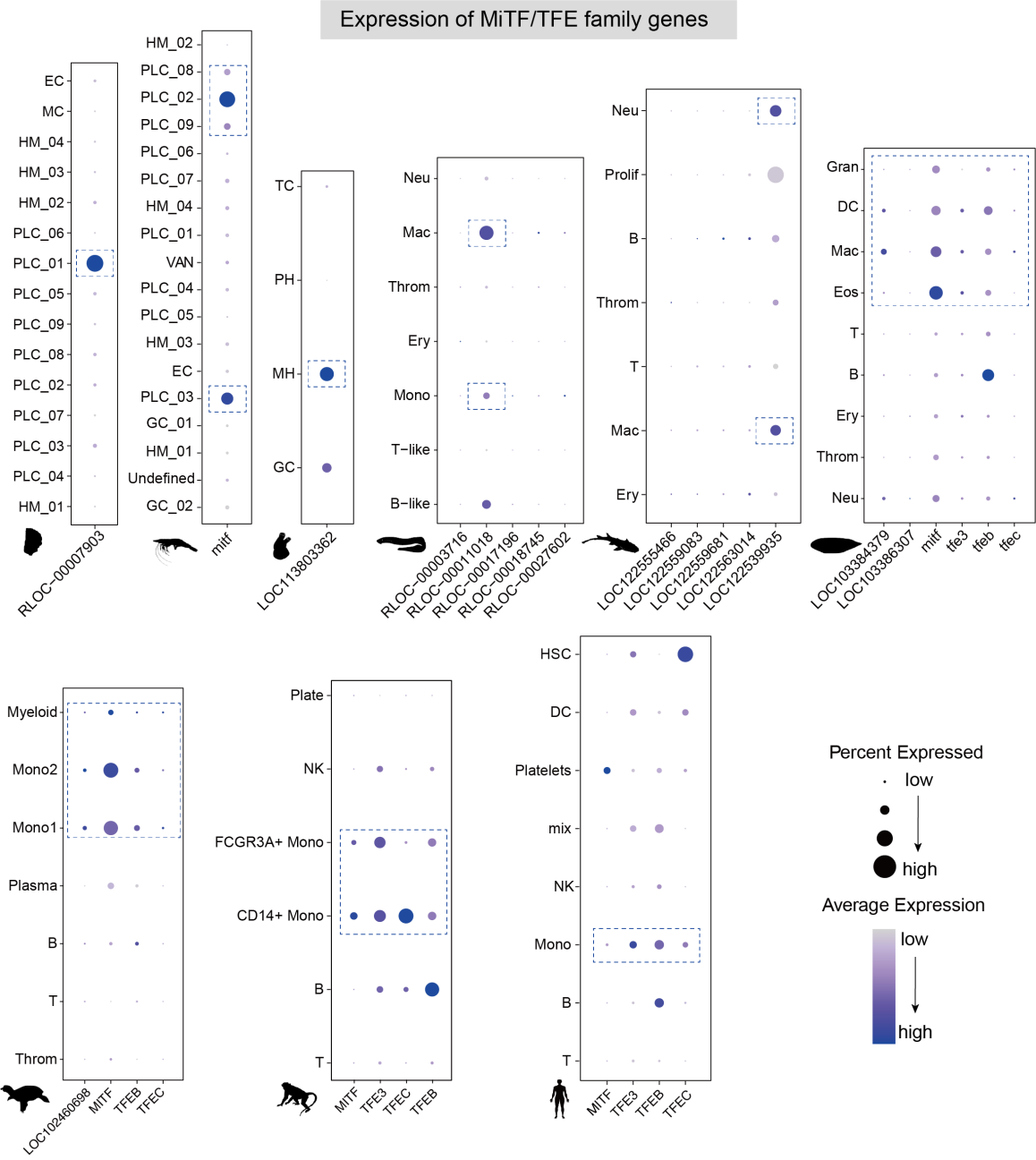


**Figure S15. Expression of the MIF/TFE gene family across species.**


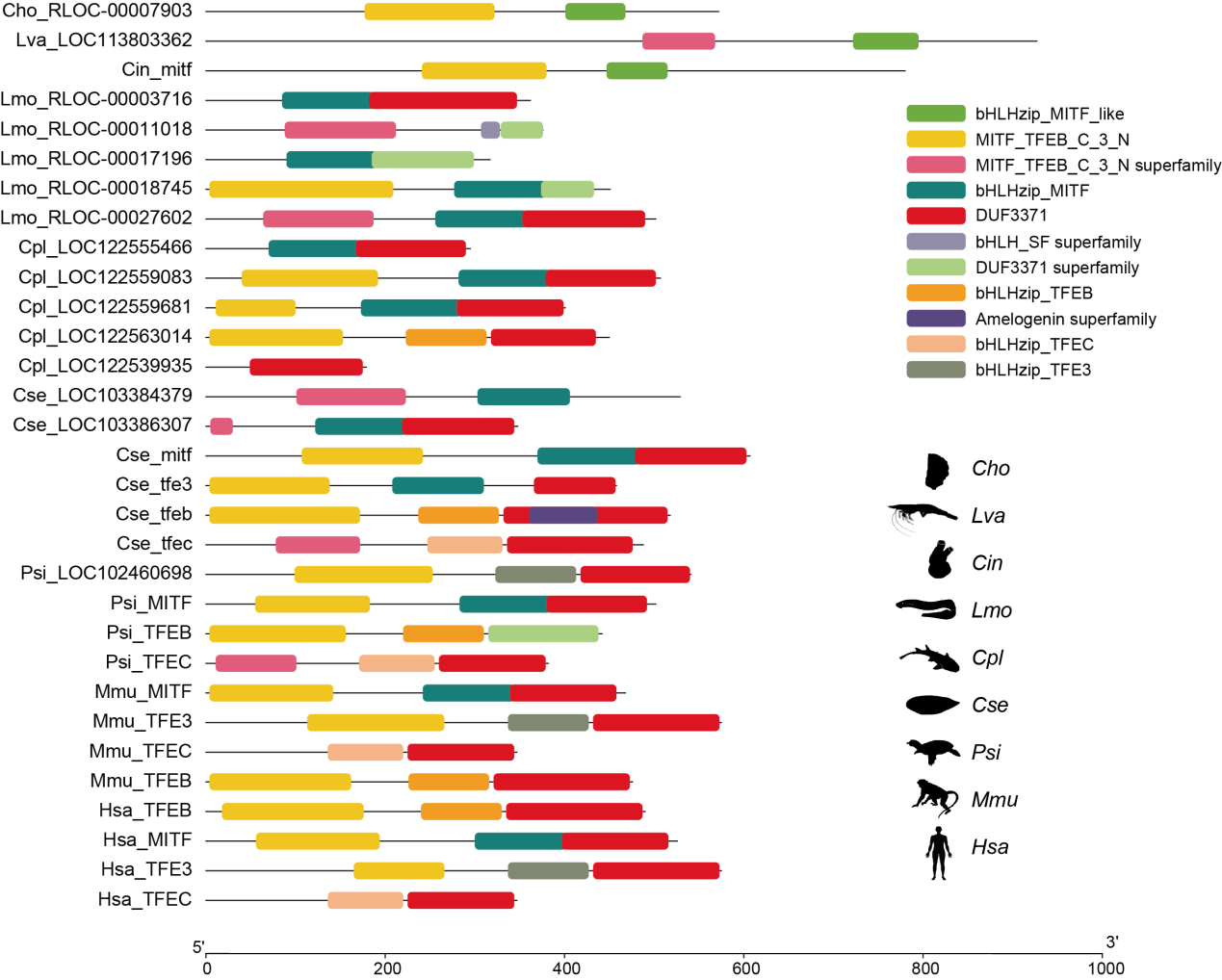


**Figure S16. The structural domains of MIF/TFE family members across species.**


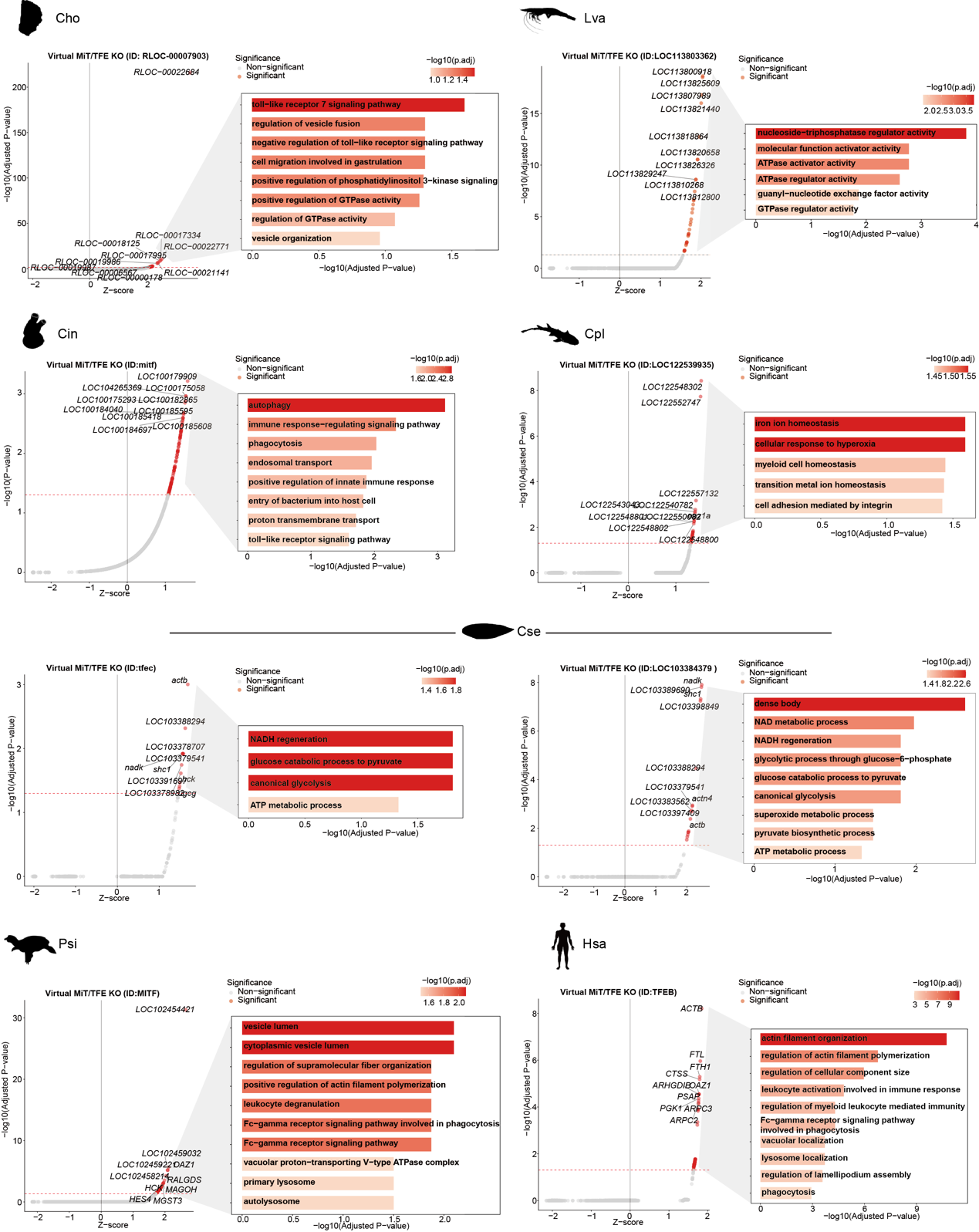


**Figure S17. Virtual knockout analysis of MiT/TFE family members across species.** Volcano plots illustrate the differentially regulated genes (DRGs, highlighted in red; Padj < 0.05) following virtual MiT/TFE perturbation in seven representative taxa, accompanied by their respective GO functional enrichment profiles.


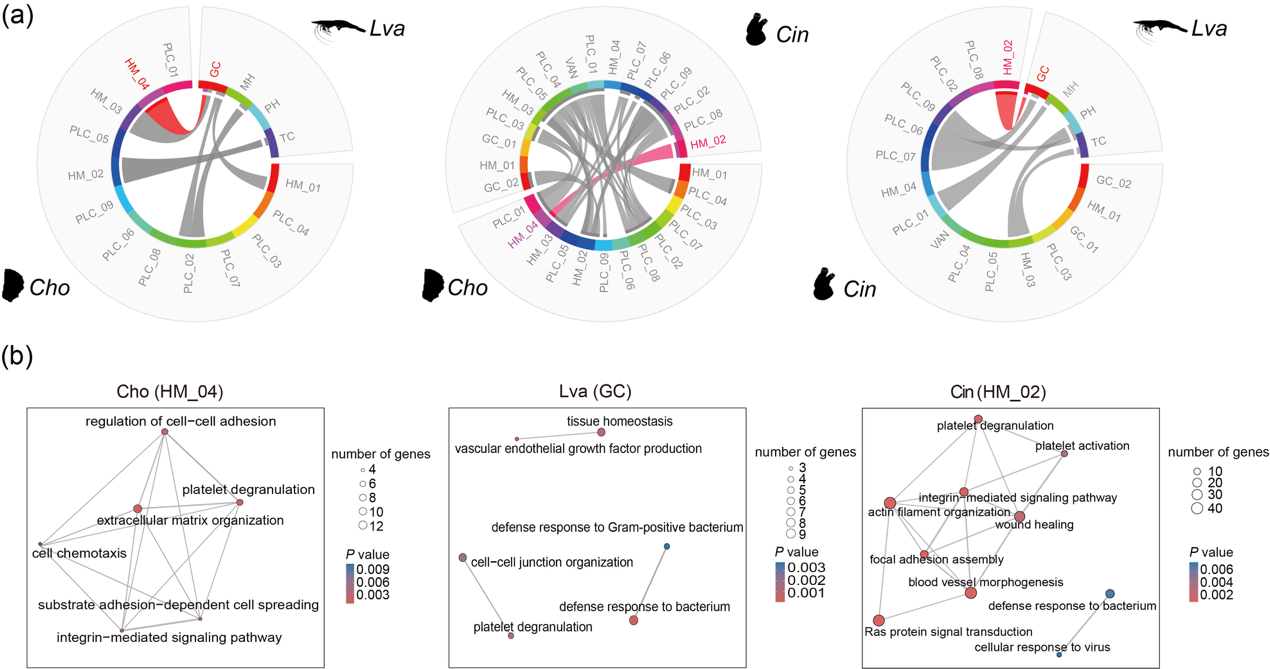


**Figure S18. Cross-species transcriptomic alignment and functional enrichment of invertebrate hemocytes.** (a) Transcriptional similarity of hemocyte populations between oysters, shrimp, and tunicate. Connections with KLD values above 88% are shown as arches linking pairs of cell types. Gray arcs denote non-coagulocytes populations. (b) GO enrichment analysis of DEGs in oyster (HM_04), shrimp (GC), and tunicate (HM_02). Some significantly enriched terms (p < 0.05) are displayed, with dot size representing gene count and color indicating p-value.


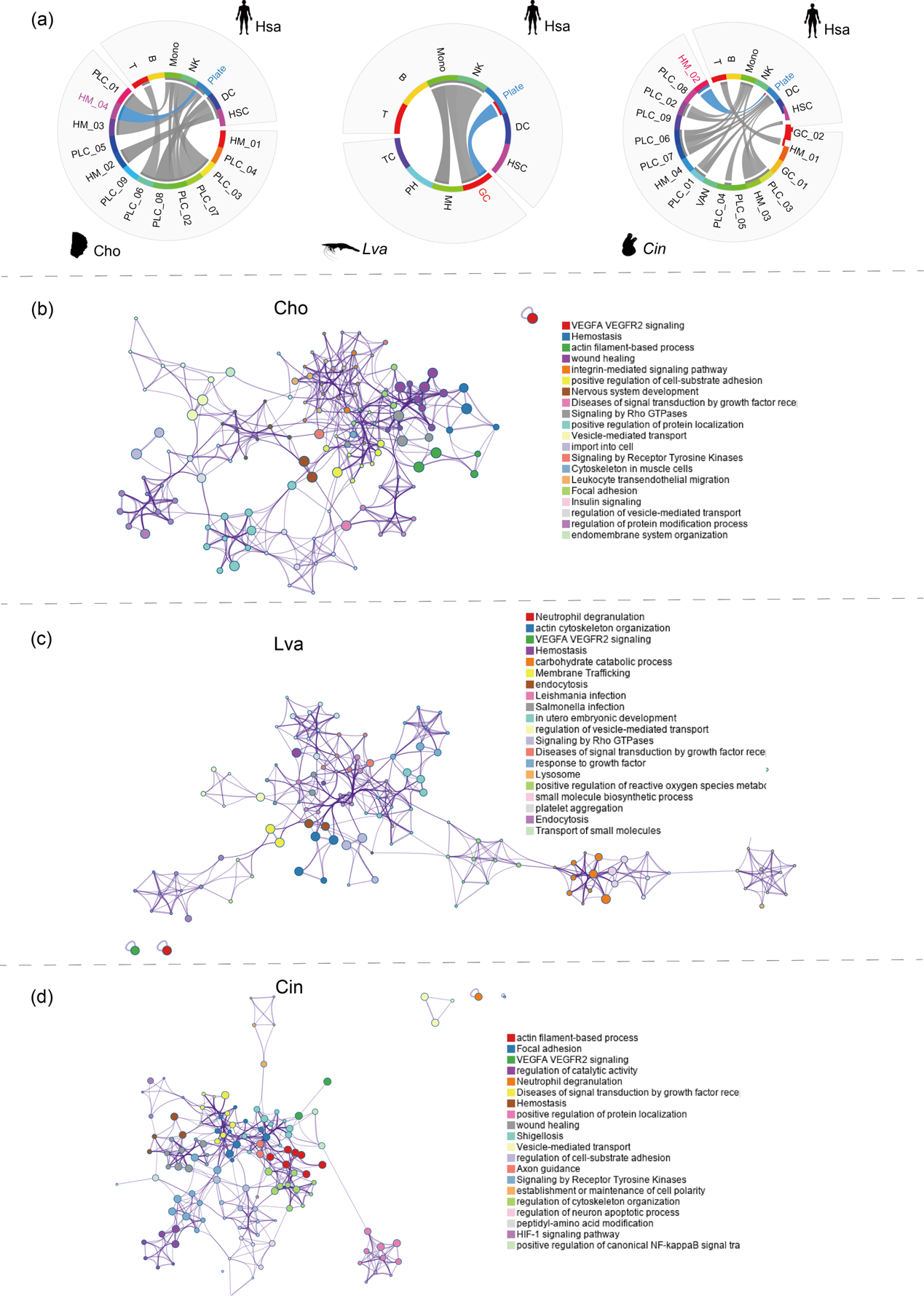


**Figure S19. Transcriptomic similarities between invertebrate coagulocytes and human platelets.** (a) Cross-species transcriptomic similarities between human blood lineages and hemocytes from the oyster, shrimp, and tunicate. Gray arcs denote non-coagulocyte populations. (b–d) Functional enrichment analysis of upregulated genes shared between human platelets and the respective coagulocytes of the oyster (b), shrimp (c), and tunicate (d).


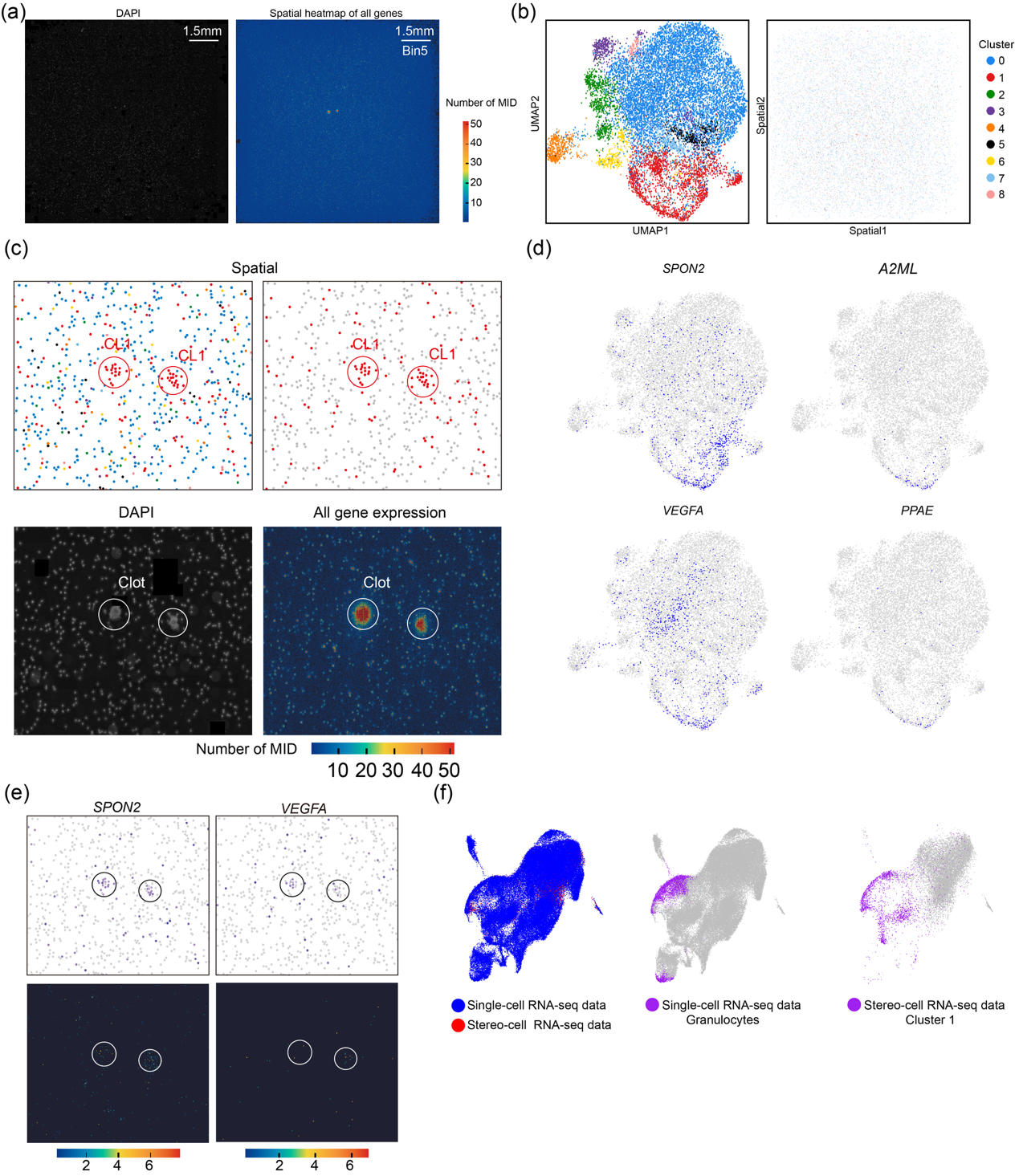


**Figure S20. Identification of cell populations with clotting function in shrimp hemocyte.** (a) Heatmap showing spatial gene expression at bin 5 resolution in shrimp (*L. vannamei*) hemocyte. Nuclei were visualized using DAPI (white). Scale bar: 1.5mm. (b) UMAP plot (left) and spatial visualization (right) of shrimp hemocyte cell populations based on gene expression. (c) Top, spatial-resolved Cluster 1 (CL; see b,) is found adjacent to blood clots (Clot). Bottom-left, DAPI staining. Bottom-right, spatial gene expression of Cluster 1. (d) UMAP plot showing the marker genes specifically expressed in Cluster 1. (e) Spatial expression of clotting genes (*SPON2* and *VEGFA*). (f) UMAP visualization of integrated of scRNA-seq and Stereo-cell in shrimp hemocyte.


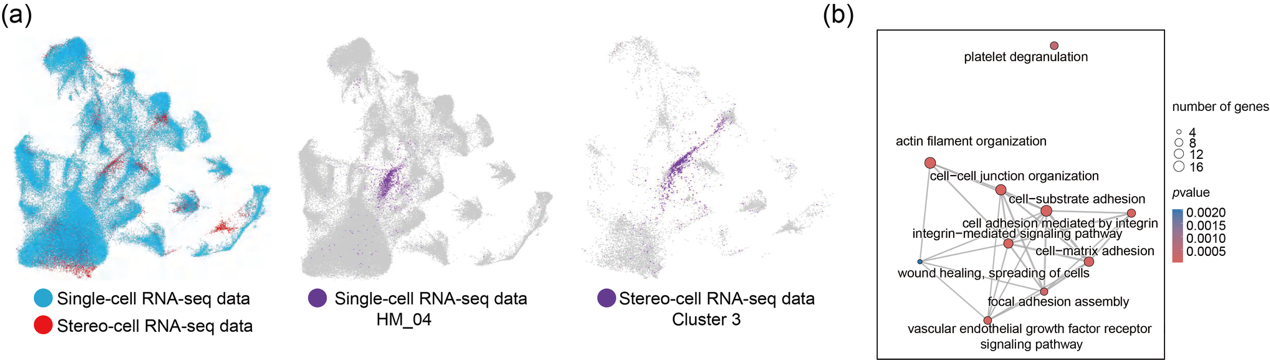


**Figure S21. Cross-platform integration and functional conservation of homologous oyster hemocytes.** (a) UMAP visualization of the integrated hemocyte datasets from *C. hongkongensis* and *C. gigas*. The scRNA-seq cluster HM_04 of *C. hongkongensis* and the Stereo-cell cluster 3 of *C. gigas* are highlighted in purple to indicate their transcriptomic homology. (b) Network plot showing the GO enrichment analysis of conserved DEGs shared between the two homologous clusters highlighted in (a). Nodes representing enriched terms are color-coded according to their adjusted *p*-values.

**Table S1. Quality information of scRNA-seq library.**

**Table S2. Differentially expressed genes (DEGs) in oyster hemocyte populations.**

**Table S3. DEGs in shrimp hemocyte populations.**

**Table S4. DEGs in tunicate hemocyte populations.**

**Table S5. DEGs in lamprey blood cell populations.**

**Table S6. DEGs in shark blood cell populations.**

**Table S7. DEGs in tonguefish blood cell populations.**

**Table S8. Marker genes for oyster hemocyte annotation.**

**Table S9. Marker genes for tunicate hemocyte annotation.**

**Table S10. Gene list for the GO term "phagocytosis" in the oyster and tunicate.**

**Table S11. Marker genes for shrimp hemocyte annotation.**

**Table S12. 7138 orthogroups shared by six vertebrates.**

**Table S13-1. 4455 orthogroups shared across all nine species.**

**Table S13-2. 4455 Ortho-genes shared across all nine species.**

**Table S14-1. The 53 orthogroups conserved in phagocytes across species.**

**Table S14-2. The 53 Ortho-genes conserved in phagocytes across species.**

**Table S15. Ancestral state reconstruction of phagocyte-active genes.**

**Table S16. List of genes within co-expression modules for each species.**

**Table S17. Clotting related genes expressed in shrimp, oysters, and tunicates.**

**Table S18. Probe information for fluorescence *in situ* hybridization experiments.**
